# DNA Barcoding the Heliothinae (Lepidoptera: Noctuidae) of Australia and Utility of DNA Barcodes for Pest Identification in Helicoverpa and Relatives

**DOI:** 10.1371/journal.pone.0160895

**Published:** 2016-08-10

**Authors:** Andrew Mitchell, David Gopurenko

**Affiliations:** 1 Australian Museum Research Institute, Australian Museum, Sydney, NSW, Australia; 2 NSW Department of Primary Industries, Wagga Wagga, NSW, Australia; 3 Graham Centre for Agricultural Innovation, Wagga Wagga, NSW, Australia; Natural Resources Canada, CANADA

## Abstract

*Helicoverpa* and *Heliothis* species include some of the world’s most significant crop pests, causing billions of dollars of losses globally. As such, a number are regulated quarantine species. For quarantine agencies, the most crucial issue is distinguishing native species from exotics, yet even this task is often not feasible because of poorly known local faunas and the difficulties of identifying closely related species, especially the immature stages. DNA barcoding is a scalable molecular diagnostic method that could provide the solution to this problem, however there has been no large-scale test of the efficacy of DNA barcodes for identifying the Heliothinae of any region of the world to date. This study fills that gap by DNA barcoding the entire heliothine moth fauna of Australia, bar one rare species, and comparing results with existing public domain resources. We find that DNA barcodes provide robust discrimination of all of the major pest species sampled, but poor discrimination of Australian *Heliocheilus* species, and we discuss ways to improve the use of DNA barcodes for identification of pests.

## Introduction

The Heliothinae is a cosmopolitan subfamily of Noctuidae containing some 365 described species worldwide [1]. The larvae feed on flowers and fruits of herbaceous plants and include a number of the world's worst agricultural pests, such as *Heliothis* and *Helicoverpa* species. Pest control is dependent on rapid and accurate species identification, however, closely related species of Heliothinae may be impossible to distinguish without genitalic dissections. While the phylogeny of Heliothinae has been relatively well studied using both molecules and morphology [2, 3, 4, 5, 6], providing a robust framework for research on heliothine genomics and biology, species diagnostics has lagged behind. Identifying *Helicoverpa* species, for example, is at best a highly specialized task, and all too often impossible for the immature stages. The obvious solution is DNA-based diagnostics, developed within a rigorous taxonomic framework. Published molecular diagnostic studies of heliothine pests have each focussed only on pairs of species. This is surprising given the global economic importance of this group and its diversity of species of agricultural significance which are found in six genera.

*Helicoverpa armigera* Hübner (1809), known as the Cotton Bollworm in Australia and as the Old World Bollworm in the Americas, was estimated to cost the Australian cotton industry $225 million annually [7] despite the successful introduction of transgenic cotton engineered to resist this pest in Australia ten years prior. *H*. *armigera* is highly polyphagous and has been recorded from around 50 different families of plants and many major crops including cotton, soybeans, corn, tobacco, tomatoes and others. Adults of *H*. *armigera* are morphologically very similar to *H*. *zea* (Boddie 1850) which is its likely sister species [5]. There are no known morphological characters to separate larvae of these species [8] and consequently there have been no resources for non-specialist identification of these species, until very recently. What prompted recent research in this area was the detection of *H*. *armigera* in Brazil, however the species seems to have been present in the country for some time when it was detected, as it had spread widely and was reported to have reduced crop yields in the 2012–2013 season by 35%, causing $1 billion in damage [9]. This is a timely reminder of the need for species diagnostic methods that work on all life stages. Clearly there remains a global need for molecular diagnostic methods that can reliably distinguish species of *Helicoverpa* and other heliothine pests.

Modern systematics studies of heliothines began with the morphological work of Hardwick [10], refined by Matthews [2], Poole [11] and others. Matthews [1] revised the Australian heliothine fauna, describing eight new species. Cho et al. [4] initiated molecular systematics studies of the higher-level phylogeny of the group, culminating in a study based on DNA sequence data for two nuclear genes (EF-1α and DDC) and one mitochondrial gene (COI), resolving many of the outstanding questions in heliothine higher-level systematics [5]. Diagnosis and identification of heliothine species is less well developed, despite their enormous economic importance, as many of the species are difficult to distinguish, there being little morphological variation among species, especially for larvae. This is particularly true of Australian *Heliocheilus* to the extent that “it is not possible to distinguish the species on the basis of the male genitalia” (Matthews 1999) and identifications require a series of specimens.

In addition to 24 species of *Heliocheilus*, the Australian Heliothinae include two species of *Adisura*, three *Australothis*, three *Heliothis* and five of the world’s approximately 20 species of *Helicoverpa*, for a total of 37 species. Three Australian *Helicoverpa* species are pests, including *H*. *armigera*, the Native Budworm *H*. *punctigera* (Wallengren, 1860) and the Oriental Tobacco Budworm *H*. *assulta* (Guenée, 1852) while *Heliothis punctifera* (Walker 1857) is a polyphagous minor pest species. *Australothis rubrescens* (Walker 1858) has a broad diet which overlaps with *Helicoverpa* species and its larvae may be confused with *Helicoverpa*. Other pest heliothines around the world include *H*. *zea* (Boddie, 1850), *H*. *gelotopoeon* (Dyar, 1921), *Chloridea virescens* (Fabricius, 1777) (previously *Heliothis virescens*, but the genus *Chloridea* was reinstated [6]) and various species of *Adisura*, *Heliothis*, *Heliocheilus* and *Masalia*.

DNA barcoding could provide an efficient way to identify heliothine species but it remains to be tested and implemented in a comprehensive manner. Previous studies developing DNA sequence data for *Helicoverpa* species have focussed on local needs, distinguishing usually just two species although sometimes with up to two other species included as outgroups [9, 12, 13, 14, 15, 16, 17, 18, 19]. On the other hand, Cho et al. [5] sequenced the DNA barcode region of the COI gene for some 70 heliothine species, including about 10 pests, however they sequenced only a single individual for most species as their aim was a phylogenetic analysis of species, not species delimitation and diagnostics. Furthermore, none of the above studies produced data that complies with the “BARCODE” data standard, which requires deposition of voucher specimens in a collection, archiving of raw sequence trace files, and sequence length and quality standards. This study aimed to fill that gap, building a comprehensive DNA barcode data set to aid identification of Australian heliothines and testing the utility of DNA barcoding for quarantine identifications.

## Materials and Methods

We first present DNA barcode data obtained from decades-old museum specimens of Australian heliothines, then add published data from GenBank to examine the utility of DNA barcoding for identification of both Australian heliothines and exotic species, particularly those of quarantine significance.

### DNA barcoding

Collecting fresh specimens for a DNA study would have been an expensive and time consuming proposition since many of these species are distributed across the remote and relatively inaccessible arid zones of northern and central Australia. To circumvent both this difficulty and the challenging task of identifying freshly collected specimens, it was decided instead to build a core data set for Australian Heliothinae using only material examined by Matthews [1]. These specimens are housed in the Australian National Insect Collection (ANIC) and most were collected in the 1990s. Four additional specimens of *Australothis tertia* collected in 2000–2003 and not examined by Matthews [1] were also sampled. In total there were 139 specimens, with mean and median ages at the time of DNA extraction of 18.3 and 16 years, respectively (see Table 1). Given the age of these specimens this required the development of a PCR primer set and amplification strategy that would allow the routine DNA barcoding of decades-old insect specimens [20].

**Table 1:** Specimens utilized for DNA barcode analysis (Data set 1).

Species	Museum Catalogue No.	Sample ID	Process ID	GenBank Accession	Sequence Length
*Adisura litarga*	31–010033	ww03053	HELAU001-15	KX422483	279
*Adisura litarga*	31–010036	ww03054	HELAU002-15	KX422482	278
*Adisura litarga*	31–010034	ww03055	HELAU003-15	KP688427	559
*Adisura litarga*	31–010719	ww03056	HELAU004-15	KP688428	559
*Adisura marginalis*	31–005657	ww03057	HELAU005-15	KP688429	559
*Adisura marginalis*	31–005638	ww03058	HELAU006-15	KP688430	559
*Adisura marginalis*		ww03059	HELAU007-15	KP688431	559
*Adisura marginalis*	31–005636	ww03060	HELAU008-15	KP688432	559
*Adisura marginalis*	31–010500	ww04854	HELAU136-15	KX422484	559
*Australothis exopisso*		ww03149	HELAU097-15	KX422488	517
*Australothis exopisso*	31–010182	ww03150	HELAU098-15	KX422490	304
*Australothis exopisso*	31–010181	ww03151	HELAU099-15	KX422489	227 [1n]
*Australothis exopisso*		ww06417	HELAU133-15	KX422485	517
*Australothis exopisso*		ww06418	HELAU134-15	KX422486	559
*Australothis exopisso*		ww06419	HELAU135-15	KX422487	559
*Australothis rubrescens*	31–013434	ww03152	HELAU100-15	KP688433	559
*Australothis rubrescens*	31–013857	ww03153	HELAU101-15	KP688434	559
*Australothis rubrescens*	31–013863	ww03154	HELAU102-15	KP688435	540
*Australothis tertia*	31–010184	ww03155	HELAU103-15		0
*Australothis tertia*	31–010183	ww03156	HELAU104-15	KX422491	304
*Australothis volatilis*	31–010608	ww03157	HELAU105-15	KX422493	559
*Australothis volatilis*	31–010609	ww03158	HELAU106-15	KX422492	559
*Helicoverpa armigera*	31–010450	ww04874	HELAU127-15	KX422494	559 [1n]
*Helicoverpa armigera*	31–013589	ww04878	HELAU137-15	KX422495	298
*Helicoverpa armigera*	31–011802	ww05394	HELAU131-15	KX422496	271
*Helicoverpa assulta*	31–010472	ww03159	HELAU107-15	KX422497	559
*Helicoverpa assulta*	31–011303	ww03160	HELAU108-15	KX422499	559
*Helicoverpa assulta*	31–011312	ww04875	HELAU130-15	KX422498	307
*Helicoverpa hardwicki*	31–010515	ww03164	HELAU112-15	KX422504	559
*Helicoverpa hardwicki*	31–010555	ww03165	HELAU113-15	KX422500	559
*Helicoverpa hardwicki*	31–010547	ww03166	HELAU114-15	KX422501	559 [1n]
*Helicoverpa hardwicki*	USNM 255832	am00959	HELAU138-16	KX422503	559
*Helicoverpa hardwicki*	USNM 255833	am00960	HELAU139-16	KX422502	363
*Helicoverpa prepodes*	31–010208	ww03161	HELAU109-15		0
*Helicoverpa prepodes*	31–010197	ww03162	HELAU110-15	KX422505	273
*Helicoverpa prepodes*		ww03163	HELAU111-15	KX422506	559
*Helicoverpa punctigera*	31–011650	ww03167	HELAU115-15	KX422508	532 [1n]
*Helicoverpa punctigera*	31–010429	ww03168	HELAU116-15	KX422509	559
*Helicoverpa punctigera*	31–013572	ww05397	HELAU132-15	KX422507	528 [2n]
*Heliocheilus abaccheutus*	31–010212	ww03065	HELAU013-15	KX422510	559
*Heliocheilus abaccheutus*	31–010211	ww03066	HELAU014-15	KX422512	559
*Heliocheilus abaccheutus*	31–010263	ww03067	HELAU015-15	KX422511	559
*Heliocheilus aberrans*	31–005797	ww03068	HELAU016-15	KX422516	559
*Heliocheilus aberrans*	31–005777	ww03069	HELAU017-15	KX422515	559
*Heliocheilus aberrans*	31–005798	ww03070	HELAU018-15	KX422513	559
*Heliocheilus aberrans*	31–005811	ww03071	HELAU019-15	KX422514	559
*Heliocheilus albivenata*	31–003794	ww03072	HELAU020-15	KX422520	559
*Heliocheilus albivenata*	31–005790	ww03073	HELAU021-15	KX422521	559
*Heliocheilus albivenata*	31–005791	ww03074	HELAU022-15	KX422517	559
*Heliocheilus albivenata*	31–005790	ww03075	HELAU023-15	KX422518	559
*Heliocheilus albivenata*	31–005790	ww03076	HELAU024-15	KX422519	559
*Heliocheilus aleurota*	31–005700	ww03077	HELAU025-15	KX422523	559
*Heliocheilus aleurota*	31–005696	ww03078	HELAU026-15	KX422524	559
*Heliocheilus aleurota*	31–005735	ww03079	HELAU027-15	KX422522	559
*Heliocheilus atrilinea*	31–013200	ww03080	HELAU028-15	KX422526	559
*Heliocheilus atrilinea*	31–013195	ww03081	HELAU029-15	KX422527	517
*Heliocheilus atrilinea*	31–013191	ww03082	HELAU030-15	KX422525	559
*Heliocheilus canusina*	31–007881	ww03083	HELAU031-15	KX422529	559
*Heliocheilus canusina*	31–010350	ww03084	HELAU032-15	KX422528	559
*Heliocheilus canusina*	31–007878	ww03085	HELAU033-15		0
*Heliocheilus cistella*	31–005953	ww03086	HELAU034-15	KX422530	559
*Heliocheilus cistella*	31–005955	ww03087	HELAU035-15	KX422531	559
*Heliocheilus cistella*	31–005931	ww03088	HELAU036-15	KX422532	559
*Heliocheilus cladotus*	31–010109	ww03089	HELAU037-15	KX422533	559
*Heliocheilus cladotus*	31–010130	ww03090	HELAU038-15	KX422535	559
*Heliocheilus cladotus*	31–010127	ww03091	HELAU039-15	KX422534	559
*Heliocheilus cladotus*	31–010108	ww03092	HELAU040-15	KX422536	559
*Heliocheilus confundens*	31–009188	ww04869	HELAU119-15	KX422538	559
*Heliocheilus confundens*	31–009192	ww04870	HELAU120-15	KX422537	559
*Heliocheilus confundens*	31–009192	ww04872	HELAU121-15	KX422541	559
*Heliocheilus cramboides*	31–006168	ww03093	HELAU041-15	KX422543	559
*Heliocheilus cramboides*	31–006155	ww03094	HELAU042-15	KX422544	559
*Heliocheilus cramboides*	31–006186	ww03095	HELAU043-15	KX422539	559
*Heliocheilus cramboides*	31–006143	ww03096	HELAU044-15	KX422540	559
*Heliocheilus cramboides*	31–006189	ww04873	HELAU118-15	KX422542	517
*Heliocheilus eodora*	31–010373	ww03097	HELAU045-15	KX422547	559
*Heliocheilus eodora*	31–010372	ww03098	HELAU046-15	KX422548	559
*Heliocheilus eodora*	31–005910	ww03099	HELAU047-15	KX422546	559
*Heliocheilus eodora*	31–005908	ww03100	HELAU048-15	KX422545	559
Heliocheilus ferruginosa*	31–007790	ww03101	HELAU049-15	KX422555	559
Heliocheilus ferruginosa*	31–007812	ww03102	HELAU050-15	KX422554	559
Heliocheilus ferruginosa*	31–007814	ww03103	HELAU051-15	KX422549	298
Heliocheilus ferruginosa*	31–007822	ww03104	HELAU052-15	KX422553	559
*Heliocheilus ferruginosa*	31–007790	ww03110	HELAU058-15	KX422552	559
*Heliocheilus ferruginosa*	31–007789	ww03111	HELAU059-15	KX422551	298
*Heliocheilus ferruginosa*	31–007817	ww03112	HELAU060-15	KX422550	559
*Heliocheilus flavitincta*	31–010636	ww03113	HELAU061-15	KX422556	517
*Heliocheilus flavitincta*		ww03114	HELAU062-15	KX422560	559
*Heliocheilus flavitincta*		ww03115	HELAU063-15	KX422558	559
*Heliocheilus flavitincta*	31–010637	ww06422	HELAU128-15	KX422557	517
*Heliocheilus flavitincta*	31–010637	ww06423	HELAU129-15	KX422559	559
*Heliocheilus halimolimnus*	31–010217	ww03105	HELAU053-15	KX422561	298
*Heliocheilus halimolimnus*	31–010222	ww03106	HELAU054-15	KX422563	559
*Heliocheilus halimolimnus*	31–010222	ww03107	HELAU055-15	KX422562	298
*Heliocheilus ionola*	31–005831	ww03108	HELAU056-15		0
*Heliocheilus ionola*	31–005792	ww03109	HELAU057-15	KX422567	298
*Heliocheilus ionola*	31–005826	ww03116	HELAU064-15	KX422564	298
*Heliocheilus ionola*	31–005832	ww06421	HELAU117-15	KX422565	489
*Heliocheilus ionola*	31–009307	ww03141	HELAU089-15	KX422566	559
*Heliocheilus melibaphes*	31–005989	ww03117	HELAU065-15	KX422570	559
*Heliocheilus melibaphes*	31–006020	ww03118	HELAU066-15	KX422569	559
*Heliocheilus melibaphes*	31–006054	ww03119	HELAU067-15	KX422568	559
*Heliocheilus mesoleuca*	31–013071	ww03120	HELAU068-15	KX422574	559
*Heliocheilus mesoleuca*	31–013082	ww03121	HELAU069-15	KX422571	559
*Heliocheilus mesoleuca*	31–013056	ww03122	HELAU070-15	KX422572	298
*Heliocheilus mesoleuca*	31–013061	ww03123	HELAU071-15	KX422573	559
*Heliocheilus moribunda*	31–005973	ww03124	HELAU072-15	KX422578	298
*Heliocheilus moribunda*	31–005975	ww03125	HELAU073-15	KX422577	559
*Heliocheilus moribunda*	31–005972	ww03126	HELAU074-15	KX422576	559
*Heliocheilus moribunda*	31–005962	ww03127	HELAU075-15	KX422575	559
*Heliocheilus neurota*	31–005865	ww03128	HELAU076-15	KX422579	559
*Heliocheilus neurota*	31–005881	ww03129	HELAU077-15	KX422580	469 [1n]
*Heliocheilus neurota*	31–010285	ww03130	HELAU078-15	KX422581	298
*Heliocheilus neurota*	31–005834	ww03131	HELAU079-15	KX422582	291
*Heliocheilus pallida*	31–006077	ww03132	HELAU080-15	KX422585	298
*Heliocheilus pallida*	31–006070	ww03133	HELAU081-15	KX422584	559
*Heliocheilus pallida*	31–006063	ww03134	HELAU082-15	KX422583	297
*Heliocheilus puncticulata*	31–011703	ww03135	HELAU083-15	KX422587	517 [1n]
*Heliocheilus puncticulata*	31–013321	ww03136	HELAU084-15	KX422588	559
*Heliocheilus puncticulata*	31–011702	ww03137	HELAU085-15	KX422589	559
*Heliocheilus puncticulata*	31–010396	ww04867	HELAU125-15	KX422586	298
*Heliocheilus ranalaetensis*	31–010234	ww03138	HELAU086-15	KX422591	559
*Heliocheilus ranalaetensis*	31–010231	ww03139	HELAU087-15	KX422590	559
*Heliocheilus ranalaetensis*	31–010237	ww03140	HELAU088-15	KX422592	555
*Heliocheilus rhodopolia*	31–009303	ww03142	HELAU090-15	KX422594	559
*Heliocheilus rhodopolia*	31–009303	ww04865	HELAU122-15	KX422593	559
*Heliocheilus rhodopolia*	31–009304	ww04866	HELAU123-15	KX422595	304
*Heliocheilus thelycritus*	31–007872	ww03143	HELAU091-15	KX422598	559
*Heliocheilus thelycritus*	31–007865	ww03144	HELAU092-15	KX422597	559
*Heliocheilus thelycritus*	31–010469	ww03145	HELAU093-15	KX422596	517
*Heliocheilus vulpinotatus*	31–010249	ww03146	HELAU094-15	KX422599	517
*Heliocheilus vulpinotatus*	31–010248	ww03147	HELAU095-15		0
*Heliocheilus vulpinotatus*	31–010260	ww03148	HELAU096-15		0
*Heliocheilus vulpinotatus*	31–010255	ww04868	HELAU124-15	KX422600	559
*Heliothis punctifera*	31–013514	ww03061	HELAU009-15	KX422601	559
*Heliothis punctifera*	31–013531	ww03062	HELAU010-15	KX422602	559
*Heliothis roseivena*	31–000810	ww03063	HELAU011-15	KX422604	559
*Heliothis roseivena*	31–008000	ww03064	HELAU012-15		0
*Heliothis roseivena*	31–010409	ww04863	HELAU126-15	KX422603	559

*Male specimens identified by Matthews (1999) as *H*. *ferruginosa* or *H*. *thelycritus* since males of the two species cannot be distinguished. Final identification based on DNA barcode data.

DNA extractions, polymerase chain reaction (PCR) amplifications and DNA sequencing was performed using the PCR primer set and amplification strategy described previously [20]. In short, the PCR strategy targets decades-old museum specimens, amplifying two short overlapping PCR fragments of approximately 300 bp each, that are subsequently reamplified using an internal primer on one end and the M13 primer on the other end. Together the two short fragments yield 559 bp of contiguous COI sequence within the DNA barcode region, fulfilling the requirements of the BARCODE standard [21].

### Data Analysis

Sequence trace files were assembled and consensus sequences constructed, aligned and trimmed using Geneious 7.1.9 [22]. Consensus sequences, specimen collection data, specimen images and sequence trace files were uploaded to the Barcode of Life Data System (BOLD,[23] and are available for download as public project Heliothinae of Australia (HELAU). Sequences were also submitted to GenBank as accession numbers KP688427—KP688435 and KX422482—KX422604. BOLD was also used for some analyses, including calculation of intra- versus interspecies distances.

Two data sets were constructed for phylogenetic analysis. Data set 1 comprised the 132 sequences derived for this study as described above. Data set 2 was composed of 1,553 sequences and was made by adding sequences retrieved from GenBank on 5 April 2016. The new data comprised 161 sequences derived from specimens collected in Australia and 1,260 sequences from non-Australian material. The latter comprised mostly exotic species but also species that occur in Australia, such as *Helicoverpa armigera*. The 161 Australian sequences comprised seven sequences from unpublished BOLD projects of the authors, 137 sequences produced from material also in the Australian National Insect Collection [24] and 17 earlier published sequences [5]. Sequences from the 3’-half of COI (not the DNA barcode region) were discarded, and the alignment was trimmed to 559 nt. Further sequences were eliminated after preliminary analyses suggested they were misidentified, e.g. GenBank accession JX509812.1 [25] ostensibly *Heliocheilus fervens*, clearly belongs in the Pyralidae (BIN BOLD:AAL8596) and was tagged on BOLD by author AM. The data set is provided as supplementary material in FastA format.

FABOX v. 1.4.1 [26] was used to edit sequence names. MEGA v.6.06 [27] was used to calculate Kimura 2-Parameter genetic distances (that distance model was chosen only to facilitate comparison with distances calculated by BOLD) and to test models of sequence evolution for phylogenetic analysis, the preferred model being the one with the lowest Bayesian Information Criterion (BIC) score. For data set 1 this proved to be the General Time Reversible model with Gamma-distributed rates (GTR+G), while for data set 2 this was the General Time Reversible model with Gamma-distributed rates and Invariable sites (GTR+G+I). Phylogenetic analyses utilized Maximum Likelihood (ML) methods and were performed in Geneious v.7.1.9 [22] using the plugins available for Fasttree 2 [28], PhyML 3.0 [29] and RAxML 7.2.8 [30]. Partitionfinder v.1.1.1 [31] was used to select a partitioning scheme for RAxML analysis.

Fasttree was used for preliminary analyses because of its speed, while PhyML and RAxML were used for final analyses. Fasttree analyses utilized the “pseudocounts” option recommended when alignments contain non-overlapping sequences. PhyML analyses optimized topology, branch lengths and rates, used the “BEST” topology search option and calculated “SH-like” support values. RAxML analyses used the ML search convergence criterion, implemented two data partitions: nucleotide positions 1 and 2 combined versus position 3, and performed 500 fast bootstrap replicates.

## Results

### Data set 1 (132 sequences)

We obtained DNA barcode data from 37 species, including 36 of Australia’s 37 species of Heliothinae, plus the New Zealand endemic species *Australothis volatilis* Matthews & Patrick (1998), with a mean of 3.6 sequences per species (S.D. = 1.22). The only Australian species we could not obtain DNA barcode data for was *Heliothis hoarei* Matthews (1999), known from only four specimens, with the two ANIC specimens collected in 1938 and 1956.

Of the 139 specimens sampled from Matthews’ [1] material examined we obtained COI sequence data for 132 specimens or 95% of samples (Table 1), which had a mean age at DNA extraction of 17.4 years. BARCODE standard compliant sequences (>486 nt in length, less than 3 N’s) were recovered from 107 specimens (77%), with a mean age of 16.4 years, and minimum and maximum ages of 8 and 38 years, respectively. Partial barcode sequences, with a mean length of 299 nt, were recovered from a further 25 specimens (18%), with a mean age at DNA extraction of 21.8 years, and minimum and maximum ages of 15 and 47 years, respectively. No sequence could be obtained from the remaining seven specimens sampled (5%) which had a mean age of 33 years, and minimum and maximum ages of 16 and 55 years, respectively.

**Fig 1:**
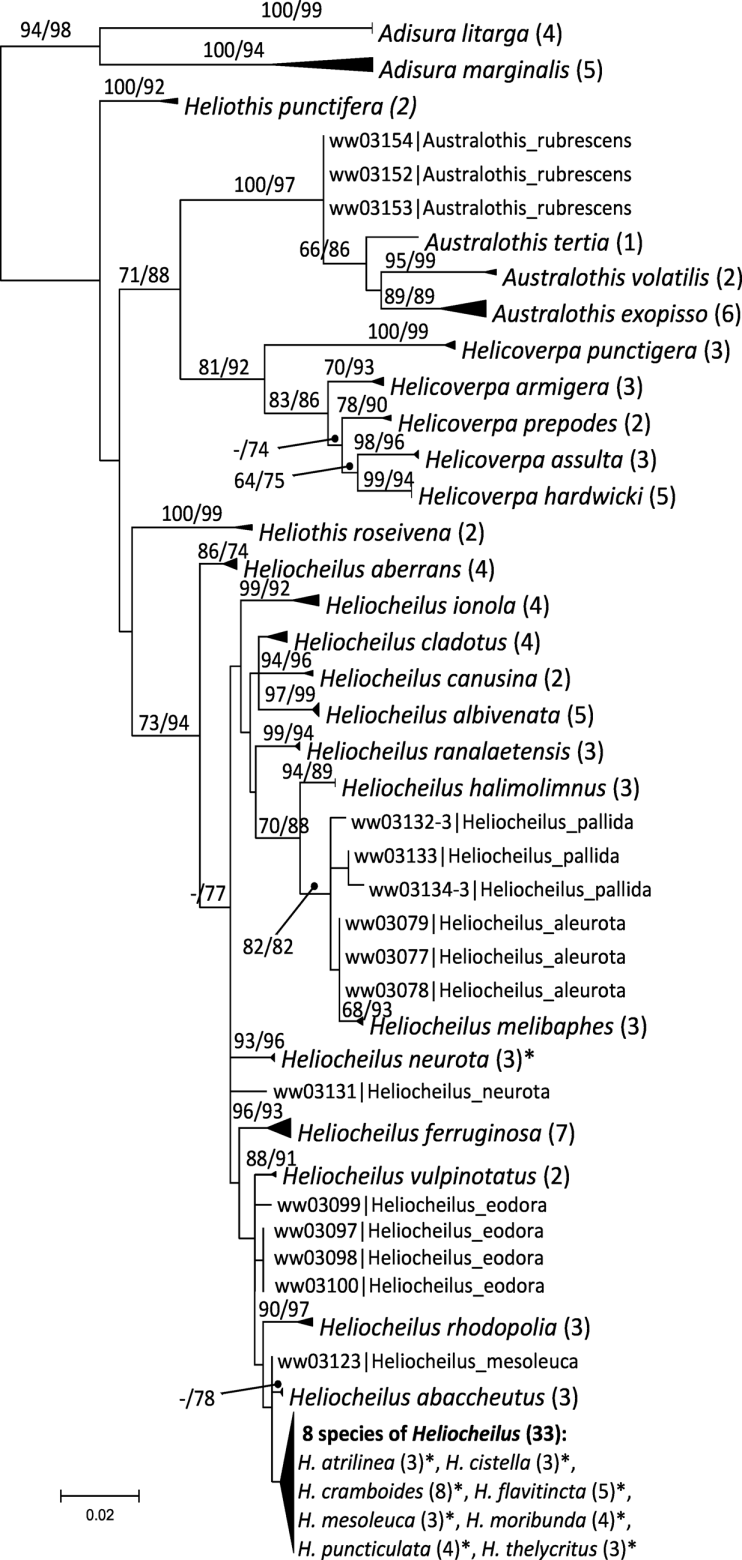
ML tree for data set 1 (132 taxa). Well-supported terminal clusters (species or species groups) collapsed. Numbers in parentheses following names are number of sequences within that group. Asterisk indicates species not recovered as monophyletic. Numbers on branches are RAxML bootstrap values followed by SH-like support values from PhyML expressed as a percentage, both shown only if ≥ 50.

The ML tree derived using PhyML is shown in Fig 1 with the species that were recovered as unique clusters collapsed to single terminal nodes (triangles). RAxML bootstrap values and ML-based SH-like support values are displayed on each branch if ≥ 0.5. All species of *Adisura* (n = 2), *Heliothis* (n = 2) and *Helicoverpa* (n = 5) were recovered as unique clusters in both ML analyses, with strong support. *Australothis* species (n = 4) were each recovered as unique clusters except for *A*. *rubrescens*. *Adisura marginalis* and *Australothis exopisso* were each divided into two distinct barcode clusters, with low levels of variation within them, but with distances of 5.1% and 4.8% between the two clusters, respectively. Other intraspecific distances were less than 1% and interspecific (nearest-neighbour) distances generally were greater than 2–3%.

For *Heliocheilus* a very different picture emerged, with only 11 of 24 species recovered as unique clusters in the PhyML analysis. Of the remaining 13 species, five were recovered as paraphyletic in one or both phylogenetic analyses (*H*. *cladotus*, *H*. *neurota*, *H*. *pallida*, *H*. *aleurota*, *H*. *eodora*) and a terminal group of eight species were recovered as a grade. Seven of the eight species in this terminal grade shared one or more haplotypes with another species. The maximum interspecific distance among the eight species peaked at 1.4%, which was roughly equal to the maximum intraspecific distance. Only one third (8 of 24) of *Heliocheilus* species displayed an obvious barcode gap (low intraspecific versus large interspecific distances).

### Data set 2 (1,553 sequences)

Data set 2 contained sequences for a number of genera not found in Australia, including the *Pyrrhia* group (*Pyrrhia*, *Heliothodes*, *Eutricopis*) (6 species), *Schinia*-group (*Schinia*, *Heliolonche*, *Psectrotarsia* and one sequence lacking identification beyond subfamily) (56 species), *Protoschinia* (1 species), *Masalia* (4 species) and *Chloridea* (2 species), the latter genus formerly known as the *Heliothis virescens* species group. Data set 2 also contained an additional 11 species and 54 sequences of *Heliothis*, five additional species and 148 sequences of *Heliocheilus* and an additional six species and 658 sequences of *Helicoverpa*, mostly from *H*. *armigera* and *H*. *zea*. Taxon sampling density for Australian species, excluding *Helicoverpa armigera*, was increased to a mean of 7.7 sequences per species (S.D. = 2.96).

**Fig 2:**
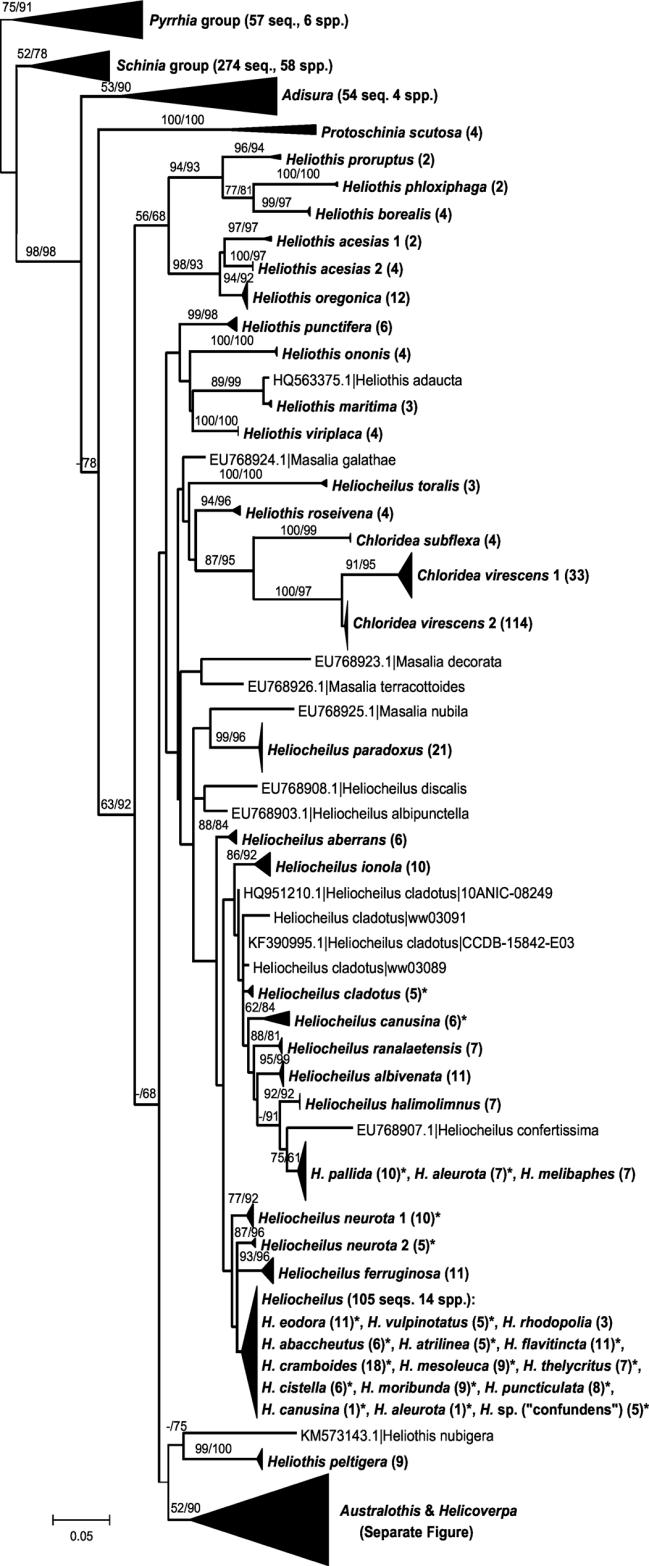
ML tree from PhyML analysis of data set 2 (1,553 sequences). Some clusters collapsed. Numbers in parentheses following names are number of sequences within that group. Numbers on branches are RAxML bootstrap values followed by SH-like support values from PhyML expressed as a percentage, both shown only if ≥ 50. Asterisk indicates species not recovered as monophyletic.

**Fig 3:**
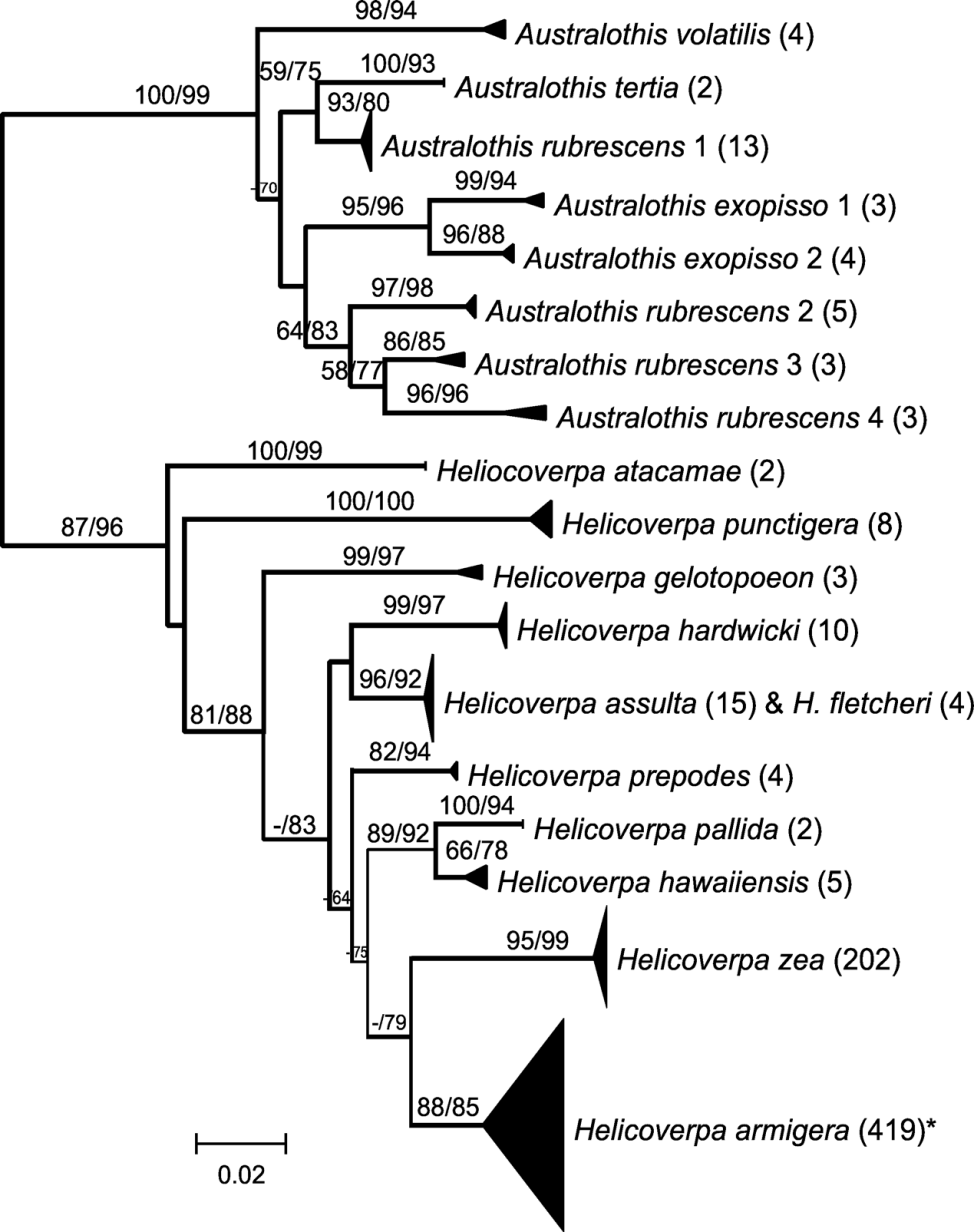
Subtree for *Australothis* and *Helicoverpa* clade, from PhyML analysis of data set 2 (1,553 sequences). Species clusters collapsed. Numbers in parentheses following names are number of sequences within that group. Numbers on branches are RAxML bootstrap values followed by SH-like support values from PhyML expressed as a percentage, both shown only if ≥ 50. Asterisk indicates species incongruence due to misidentifications (see discussion).

The full ML tree derived using PhyML for this expanded dataset including all public barcode region data for Heliothinae is shown in two parts as S1 Fig and S2 Fig. The ML tree was rooted with the entire *Pyrrhia* group [5], comprising six species in three genera. All *Pyrrhia*-group species and 39 of 42 *Schinia*-group species with multiple sequences were recovered as unique clusters. The ML tree is redrawn in Figs 2 and 3 with many single-taxon clusters collapsed to aid in visualizing species recovery for the remaining taxa.

Fig 2 shows relationships among species of *Heliothis*, *Chloridea*, *Masalia* and *Heliocheilus*. All *Heliothis* and *Chloridea* species are recovered as unique clusters, however *H*. *acesias* and *C*. *virescens* consisted of two clusters separated by > 5% and >2% sequence divergence, respectively. *Masalia* species were represented by only one sequence each, but were separated by >2% from their nearest neighbours. For *Heliocheilus*, results were comparable with data set 1, except that five exotic species were sampled, only seven of the 24 species were placed in unique clusters, and the terminal grade that contained eight species in data set 1 comprised 14 species.

Fig 3 shows relationships among *Australothis* and *Helicoverpa*. Both genera were recovered as unique clusters, with strong support. *A*. *rubrescens* samples were divided among four distinct clusters, each separated by more than 3.5% sequence divergence. All species of *Australothis* were well separated from each other but there was little support for relationships among species. Eleven species of *Helicoverpa* were represented in Fig 3 and all were recovered as unique clusters and well separated from other species, except for *H*. *assulta* and *H*. *fletcheri*, which shared haplotypes. In addition, four sequences within the *H*. *armigera* cluster, which contained 419 sequences, were identified as either *H*. *assulta* or *H*. *punctigera*.

## Discussion

### DNA barcode sequence recovery

Specimen age affected our ability to recover DNA barcode sequences. Using the PCR primers and amplification strategy developed for older specimens [20] we were able to get BARCODE standard compliant sequences (i.e. >486 nt of contiguous sequence with two or fewer ambiguous sites) from 107 of 139 (77%) samples with a mean age at DNA extraction of 18.1 years, and partial barcode data from 132 of 139 (95%) of samples.

Only two thirds of the Australian species sampled were recovered in unique barcode clusters by the ML analyses. As a result, during the early stages of this project we could not rule out the possibility of cross-contamination of samples during DNA extraction or PCR. This possibility was of concern because the PCR procedure we employed relies on reamplification of initial PCR products using hemi-nested primers [20]. However, a number of factors subsequently convinced us of the veracity of our data. The first was our use of copious negative controls in our experiments, with empty wells in tissue sample plates being subjected to DNA extraction and two rounds of PCR without detection of PCR products. A second factor was the lack of sequence variation detected for each sample in the region of overlap between the two half-length barcode fragments. Despite being only 58bp in length this region contains 3–4 highly variable sites that often differ even between closely related species. The third factor was the independent publication of DNA barcode data [24] for much of the Australian Lepidoptera fauna, including Heliothinae. Inclusion of this data [24] in our expanded data set 2 resulted in almost complete congruence, with their sequences either clustering with, or being >99% similar to, conspecific sequences in our data set 1.

### Do DNA barcodes track species boundaries?

The ML tree derived from data set 2, the expanded set of all public data for Heliothinae (Figs 2 and 3, S1 and S2 Figs) gave very similar results for Australian taxa to that presented in Fig 1. Many of the additional sequences in data set 2 were from non-Australian taxa such as *Pyrrhia*, *Schinia* and related genera. With minor exceptions all of these species were recovered as unique clusters and they were not considered further in this study. We note however that DNA barcoding is known to fail in some species of *Schinia* which were not included in our data set: DNA barcode data from 35 specimens failed to track species boundaries in the six species of the *S*. *volupia* species complex [32]. Unfortunately these sequences were not captured by our search term “Heliothinae” on GenBank because the sequences were suppressed (e.g. see GenBank accession GU702778) when their identification was not updated beyond “Lepidoptera”, thus they were not included in this study.

Increased sampling of heliothine species and greatly increased geographic sampling of some of the non-endemic Australian species, resulted in a much higher rate of species recovery in single clusters (“monophyly”) overall, despite further reducing the already poor rate of recovery of Australian *Heliocheilus* species. Below we discuss the most significant results by genus.

#### Heliocheilus

Based on our ML analyses, only one third (eight of 24) of Australian *Heliocheilus* species were recovered as unique clusters and had discernible barcode gaps between species: *H*. *aberrans*, *H*. *albivenata*, *H*. *ferruginosa*, *H*. *halimolimnus*, *H*. *ionola*, *H*. *melibaphes*, *H*. *ranalaetensis*, and *H*. *rhodopolia*. The last species was included in the terminal grade of 14 species only because the relationships among it and other species were too complex to illustrate in Fig 2 (but see S1 Fig). Four species that were recovered as unique clusters in data set 1 were placed in multiple clusters in data set 2 due to the inclusion of new haplotypes in the expanded dataset, i.e., *H*. *cladotus*, *H*. *abaccheutus*, *H*. *vulpinotatus* and *H*. *canusina*. For *H*. *cladotus*, *H*. *abaccheutus*, *H*. *vulpinotatus*, *H*. *eodora* and *H*. *pallida*, paraphyly seems to result only from the very low levels of sequence variation and a resulting lack of differentiation among species, and it is probable that sequencing a larger portion of the mitochondrial genome would provide enough informative characters to recover those species as unique clusters and distinct from closely related species. However, *H*. *aleurota* and *H*. *canusina* each had a single distant sequence (>1.8% divergent from the others for that species) added from published data [24], resulting in those species being represented in the terminal grade of 14 species in data set 2. For the latter two species we cannot exclude the possibility of cross-contamination or misidentification of samples. For *H*. *neurota* there are two distinct clusters of sequences which are up to 1.8% divergent from each other, and neither is included in the terminal grade of 14 species.

The BOLD BIN database [33] includes 19 species in a single BIN (BOLD:ACE4297) which corresponds closely to our terminal grade of 14 species (noting that species may occur in multiple BINs). Three species that we regard as diagnosable through ML-based analysis of DNA barcode data are included in this BOLD BIN, i.e., *H*. *cladotus*, *H*. *ferruginosa* and *H*. *neurota*, but this difference just reflects our differing methodologies. Other species names included in this BOLD BIN in error are: *H*. *epigrapha* (a junior synonym of *H*. *ferruginosa*), *H*. *venata* and *H*. *neurias* (both junior synonyms of *H*. *cramboides*), *H*. *clathrata* (a junior synonym of *H*. *neurota*) (Matthews 1999), *H*. *confundens* (see discussion below on *H*. *confundens*) and *Rivula niphodesma*. The latter species bears a superficial resemblance to *H*. *cramboides* but belongs in a different family, Erebidae, and is clearly a misidentified specimen.

The remaining eight species in the terminal grade are the same eight species recovered in the terminal grade of data set 1 (Fig 1). These eight species are completely intermingled in the trees, and seven of eight species share at least one haplotype with other species. Given that there is little morphological variation among Australian *Heliocheilus* species, this raises the question whether all the named species indeed warrant species status. However, it turns out that some of the most similar looking species pairs have quite distinctive DNA barcodes (e.g. *H*. *aberrans* versus *H*. *albivenata*) while some of the species sharing haplotypes with other species have very distinctive wing patterns (e.g. *H*. *cistella* and *H*. *flavitincta*). In fact, *H*. *flavitincta* is the most distinctive of all *Heliocheilus* with a yellow-orange ground colour to the forewings, overlaid by dark brown to black lines, whereas most of its congeners are a dull pale brown with indistinctive markings. It is possible that introgression is involved here, or a *Wolbachia*-mediated mtDNA selective sweep.

Many *Heliocheilus* species are difficult to differentiate from other species. *H*. *ferruginosa* males cannot be separated from *H*. *thelycritus* males as the only diagnostic characters are in the female genitalia [1]. Therefore some of the male specimens sampled were initially labelled “H. ferruginosa-thelycritus” to indicate that they could be either species. Fortunately, positively identified females of each species were well separated in the trees, with *H*. *thelycritus* being placed in the terminal group of eight species indistinguishable by barcodes but *H*. *ferruginosa* females forming a distinct group of their own. Males of the two species associated with one or the other cluster and were secondarily labelled with the appropriate species name.

*Heliocheilus confundens*, although treated in the revision of Australian Heliothinae, is known only from Indonesia [1] and was not sampled by us in this study (data set 1). Five specimens supposedly of this species were sampled in a previous study [24] and are included in BIN BOLD:ACE4297, however the specimens were collected in north-western Australia and were examined by Matthews as part of his revision, therefore they cannot be *H*. *confundens*. We treated these specimens as *Heliocheilus* sp. although we have retained the name “confundens” in parentheses in S1 Fig, however the specimens appear to us to be *H*. *cramboides*, as the photographs and collection data for some of these specimens matches those of *H*. *cramboides* specimens examined by Matthews [1].

Suitable nuclear gene data could shed light on the reasons for DNA barcode failure in Australian *Heliocheilus*, however, because all the material sequenced for this study is decades old it was not possible to sequence nuclear DNA from these specimens using conventional PCR-based Sanger sequencing approaches. We note that a previous study [5] also found low levels of variation among the Australian *Heliocheilus* species in both nuclear genes examined, and this likely represents a recent, rapid radiation.

#### *Heliothis*, *Australothis* and *Adisura*

DNA barcoding proved successful in distinguishing all species of these genera for which barcode data was obtained, including four species of *Adisura*, 12 species of *Heliothis*, given that *H*. *adaucta* is a synonym of *H*. *maritima*, and four species of *Australothis*. The two distinct sequence groups (BOLD BINs) recovered for *Adisura marginalis* and the four groups of *Australothis rubrescens* warrant further investigation as they could represent cryptic species, *Wolbachia* infected lineages, genetically diverged populations and/or ancestral mitochondrial lineages retained within populations.

#### Chloridea

Two very distinct barcode clusters were recovered for *Chloridea virescens* which is considered the second worst pest in the western hemisphere after *Helicoverpa zea* [11], and is a major pest of cotton, tobacco and soybeans. Interestingly, the 114 sequences from Brazilian specimens [34] formed a distinct group, indeed a different BOLD BIN, 2.1–3.6% distant from the remaining *C*. *virescens*, which were collected from North, Central and South America. This raises the possibility that the Brazilian populations sampled previously [34] belong to a different species and have been misidentified. Apart from *C*. *virescens* and *C*. *subflexa*, the only *Chloridea* species currently on BOLD is *C*. *molochitina*, however searching BOLD with the Brazilian sequences does not result in a BOLD match to that species. The only other species of *Chloridea* known to be a minor pest is *C*. *tergemina* [6]. The possibility that cryptic sibling species of *C*. *virescens* exist has been raised before [11] and this hould be investigated further for the specimens from Brazil [34].

#### Helicoverpa

DNA barcodes readily distinguish 10 of the 11 species of *Helicoverpa* for which barcode data exists in the public domain. The exception is sequences identified as being from *H*. *fletcheri* (GenBank accessions KF492623—KF492626) which are identical to sequences of *H*. *assulta*. A tropical African species, *H*. *fletcheri* was placed it in the *H*. *zea*-group and regarded as most similar to *H*. *toddi* [10], but *H*. *toddi* sequences were not available for comparison. These sequences were deposited in GenBank in 2013 but are not yet associated with a publication that confirms the species identifications, therefore the identity of these sequences should be treated with caution.

There are four anomalous sequences nested within the *H*. *armigera* cluster. One sequence was identified as *H*. *punctigera* in a study analysing the diets of invertebrate predators using COI sequences (GenBank accession JQ240198.1, [35]). The other three sequences (GenBank accessions JX509775 –JX509777) were identified as *H*. *assulta* in a study which concluded that *H*. *assulta* and *H*. *armigera* had identical DNA barcodes[15]. However, our data demonstrates a minimum of 2.4% divergence between the latter two species, and at least 4% distance between *H*. *punctigera* and any other species. We conclude that these sequences are almost certainly misidentified to species, however neither of the cited papers provides any information about voucher specimens or the basis on which the identifications were made. Therefore there is no way to check the identifications and the sequences should be disregarded.

The two most economically important species of *Helicoverpa* are *H*. *zea* and *H*. *armigera*, and our data set contains 202 sequences of the former species and 419 of the latter, with most of the data from published data sets [9, 12, 13, 19, 36]. The specimens were collected from 22 countries, covering most of the known distributions of both species, including North America (for *H*. *zea*), South America (for both species), Australia, Asia and Europe (for *H*. *armigera*). The two species were each recovered as unique clusters and as sister-groups, separated by a minimum genetic distance of 1.9%. Thus DNA barcoding holds up to global sampling and can be used to distinguish these species reliably.

### Identification of heliothine pest species

The incursion of *H*. *armigera* in Brazil went undetected for about five years [37] giving the species time to establish on corn, soybean and cotton and spread throughout the country, reducing crop yields by 35% and resulting in economic losses of about $1 billion [9]. Early detection of *H*. *armigera* might have prevented this biological invasion. However, distinguishing H. *armigera* and *H*. *zea* adults is a difficult and specialized task and larvae of *H*. *armigera* cannot be distinguished from those of *H*. *zea* using morphology, despite efforts to find morphological characters. For *H*. *armigera* and *H*. *zea*, the head chaetotaxy, mandibles, hypopharyngeal complex, body coloration and markings, body chaetotaxy, pinacula size and shape, setal color, cuticle texture, and crochet counts and arrangement for various instars do not bear any morphological characters that reliably separate larvae of these two species [8]. Instead a nuclear ribosomal DNA-based Real Time PCR assay based on ITS2 sequences was proposed to identify immature stages of these species [14], and a similar PCR assay based on ITS1 has also been proposed [17].

Although rapid molecular diagnostic tests now exist for distinguishing *H*. *armigera* from *H*. *zea*, there remains an urgent need for molecular diagnostics methods which can distinguish other *Helicoverpa* species, *Heliothis* species and other heliothine pest species. DNA barcodes provide an ideal platform for such identifications because there is no limit to the number of species that can be detected with a single assay. This study demonstrates that DNA barcodes also can be used to reliably distinguish the economically important species of *Helicoverpa* (with the possible exception of the minor pest *H*. *fletcheri*, which unpublished data on GenBank suggests may have identical DNA barcodes to *H*. *assaulta*), *Heliothis*, *Chloridea*, and likely most other species of Heliothinae. Australian *Heliocheilus* species are a notable exception with less than half of the species being diagnosable using DNA barcodes. Those species that cannot be diagnosed using DNA barcodes form a single cluster. In addition, none of the Australian *Heliocheilus* are pests, thus quarantine agencies using barcode data would easily be able to tell native from exotic species and pests, such as the African species *H*. *albipunctella* (the Millet Head Miner), from non-pests.

### Standards for quarantine identifications

DNA databases used for quarantine identifications require high levels of data integrity and data redundancy [38]. While BOLD is an enormously useful tool for species identification, it is not without errors resulting from incorrectly identified specimens and/or cross-contamination of samples. While there are advantages to having all the non-BARCODE compliant COI gene sequences from GenBank stored on BOLD, it can also be a source of error. It is also not unprecedented, in our experience, to encounter publically released data on BOLD that has been incorrectly identified to species. This ultimately undermines the usefulness of BOLD for applications such as quarantine identifications. We note that BOLD has a facility for community third-party annotation of barcode records, and the Barcode Index Number (BIN) system [33] provides an efficient mechanism for detection of taxonomic misassignments. However, we argue that these facilities alone are insufficient to ensure a high standard of species identifications. Instead we advocate for a second, higher barcode standard that should be applied to regulated species such as those of quarantine importance, commercial fish species, IUCN red-listed species, etc. The second standard would have more rigorous criteria for species identification, shifting the onus to data submitters to demonstrate unequivocally that their voucher specimens have been accurately identified, for example by providing photographic evidence of genitalia dissections or other necessary diagnostic characters, and/or having identifications vetted by independent taxonomic experts. Other criteria that might also been considered in the higher standard include whether all closely related species that need to be distinguished have been sampled.

## Conclusions

DNA barcodes were assembled for the entire heliothine moth fauna of Australia, bar one rare species. The data revealed deep mtDNA divergences in two Australian species, which may represent cryptic species, but very shallow divergences among about half of the Australian fauna of *Heliocheilus*, which consequently cannot be identified using this method. Of the 91 species remaining in the expanded global data set after excluding all *Heliocheilus*, 87 species (96%) were readily identifiable with DNA barcodes. Thus DNA barcoding can provide a powerful solution to quarantine identifications of *Helicoverpa*, *Heliothis* and other Heliothinae. While real time PCR methods for identifying *Helicoverpa* species are faster, current methods are useful only for distinguishing between *H*. *armigera* and *H*. *zea*, and may give misleading results if other species are processed unwittingly. Such assays are therefore best suited to high-throughput screening once identifications have been narrowed down to a few choices through other means. A much more powerful approach is to derive DNA sequence data which can be used to query an extensive database of reference sequences for many species. However, more emphasis is needed on distinguishing true “reference” sequences from others. Reference sequences should not only be of highest quality and derived from properly vouchered specimens, their species identifications should be backed by scientific data such as images of diagnostic morphological characters, and they should be performed or vetted by taxonomic experts.

## Supporting Information

S1 Fig ML tree from PhyML for data set 2 (1,553 taxa). Subtree containing *Australothis* and *Helicoverpa* collapsed (see S2 Fig).
(PDF)

S2 Fig Subtree for *Australothis* and *Helicoverpa* from ML tree from PhyML for data set 2 (1,553 taxa). (PDF)

S1 File FastA Alignment, Data Set 2, 1,553 taxa. Sequence names include either a GenBank Accession number or a BOLD Sample ID (or both for sequences from Hebert et al. 2013) in the format: “GenBank Accession number|Species name|BOLD Sample ID”.
(FAS)
