# Supplementary material for: DNA Barcoding the Heliothinae (Lepidoptera: Noctuidae) of Australia and Utility of DNA Barcodes for Pest Identification in Helicoverpa and Relatives
Source: PLoS One. 2016 Aug 10;11(8):e0160895. doi: 10.1371/journal.pone.0160895 (PMC4980029; doi:10.1371/journal.pone.0160895)
Supplement: S1 Fig — Subtree containing Australothis and Helicoverpa collapsed (see S2 Fig). (PDF) [file pone.0160895.s001.pdf]

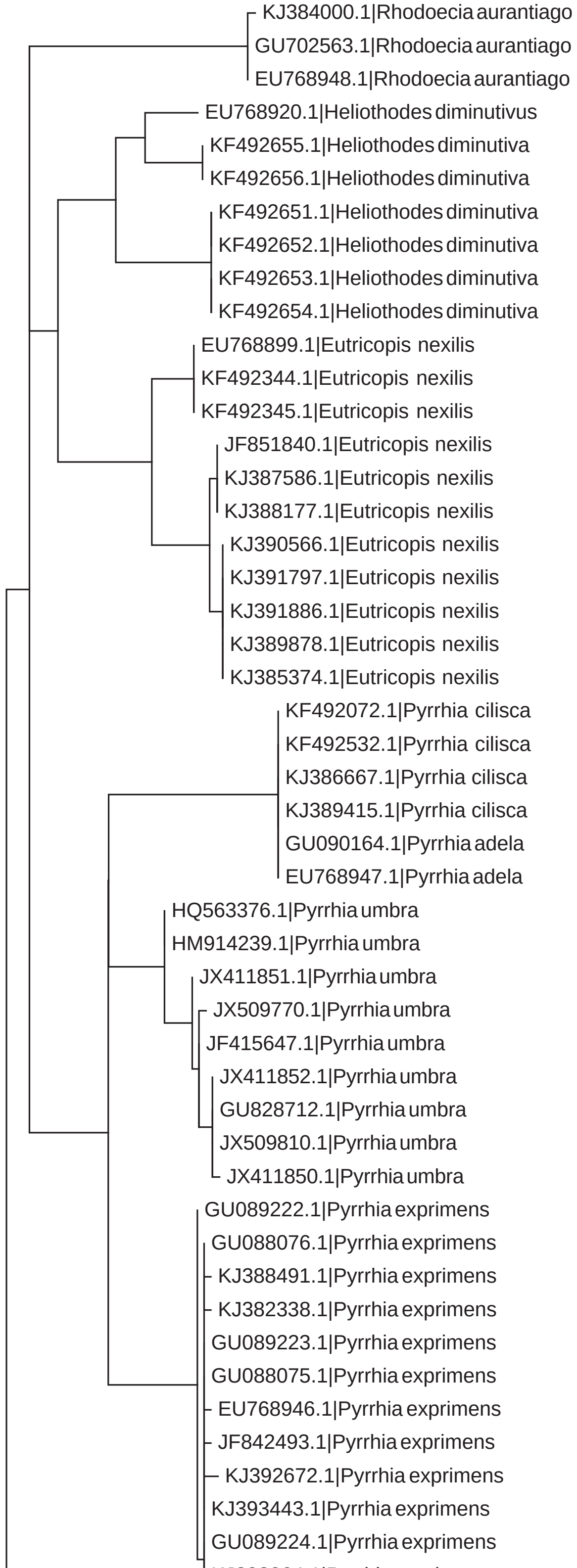

KJ384000.1|Rhodoecia aurantiago
GU702563.1|Rhodoecia aurantiago
EU768948.1|Rhodoecia aurantiago
EU768920.1|Heliothodes diminutivus
KF492655.1|Heliothodes diminutiva
KF492656.1|Heliothodes diminutiva
KF492651.1|Heliothodes diminutiva
KF492652.1|Heliothodes diminutiva
KF492653.1|Heliothodes diminutiva
KF492654.1|Heliothodes diminutiva
EU768899.1|Eutricopis nexilis
KF492344.1|Eutricopis nexilis
KF492345.1|Eutricopis nexilis
JF851840.1|Eutricopis nexilis
KJ387586.1|Eutricopis nexilis
KJ388177.1|Eutricopis nexilis
KJ390566.1|Eutricopis nexilis
KJ391797.1|Eutricopis nexilis
KJ391886.1|Eutricopis nexilis
KJ389878.1|Eutricopis nexilis
KJ385374.1|Eutricopis nexilis
KF492072.1|Pyrrhia cilisca
KF492532.1|Pyrrhia cilisca
KJ386667.1|Pyrrhia cilisca
KJ389415.1|Pyrrhia cilisca
GU090164.1|Pyrrhia adela
EU768947.1|Pyrrhia adela
HQ563376.1|Pyrrhia umbra
HM914239.1|Pyrrhia umbra
JX411851.1|Pyrrhia umbra
JX509770.1|Pyrrhia umbra
JF415647.1|Pyrrhia umbra
JX411852.1|Pyrrhia umbra
GU828712.1|Pyrrhia umbra
JX509810.1|Pyrrhia umbra
JX411850.1|Pyrrhia umbra
GU089222.1|Pyrrhia exprimens
GU088076.1|Pyrrhia exprimens
KJ388491.1|Pyrrhia exprimens
KJ382338.1|Pyrrhia exprimens
GU089223.1|Pyrrhia exprimens
GU088075.1|Pyrrhia exprimens
EU768946.1|Pyrrhia exprimens
JF842493.1|Pyrrhia exprimens
KJ392672.1|Pyrrhia exprimens
KJ393443.1|Pyrrhia exprimens
GU089224.1|Pyrrhia exprimens

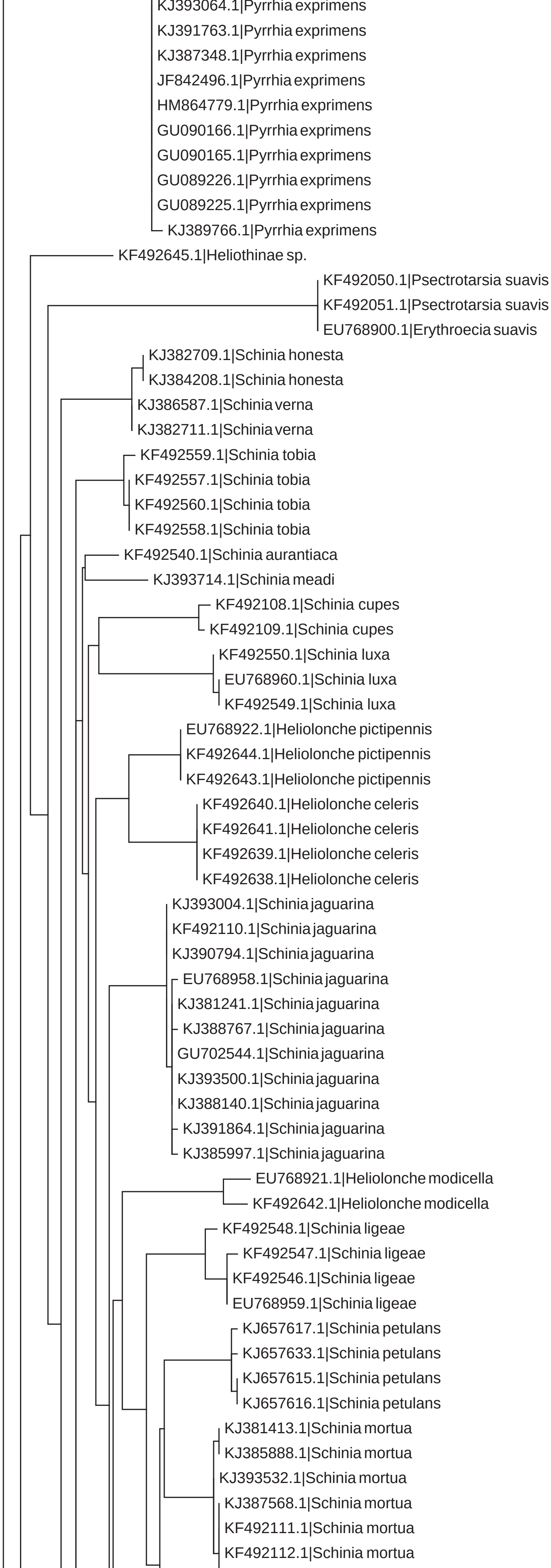
KJ393064.1|Pyrrhia exprimens
KJ391763.1|Pyrrhia exprimens
KJ387348.1|Pyrrhia exprimens
JF842496.1|Pyrrhia exprimens
HM864779.1|Pyrrhia exprimens
GU090166.1|Pyrrhia exprimens
GU090165.1|Pyrrhia exprimens
GU089226.1|Pyrrhia exprimens
GU089225.1|Pyrrhia exprimens
KJ389766.1|Pyrrhia exprimens
KF492645.1|Heliothinae sp.
KF492050.1|Psectrotarsia suavis
KF492051.1|Psectrotarsia suavis
EU768900.1|Erythroecia suavis
KJ382709.1|Schinia honesta
KJ384208.1|Schinia honesta
KJ386587.1|Schinia verna
KJ382711.1|Schinia verna
KF492559.1|Schinia tobia
KF492557.1|Schinia tobia
KF492560.1|Schinia tobia
KF492558.1|Schinia tobia
KF492540.1|Schinia aurantiaca
KJ393714.1|Schinia meadi
KF492108.1|Schinia cupes
KF492109.1|Schinia cupes
KF492550.1|Schinia luxa
EU768960.1|Schinia luxa
KF492549.1|Schinia luxa
EU768922.1|Heliolonche pictipennis
KF492644.1|Heliolonche pictipennis
KF492643.1|Heliolonche pictipennis
KF492640.1|Heliolonche celeris
KF492641.1|Heliolonche celeris
KF492639.1|Heliolonche celeris
KF492638.1|Heliolonche celeris
KJ393004.1|Schinia jaguarina
KF492110.1|Schinia jaguarina
KJ390794.1|Schinia jaguarina
EU768958.1|Schinia jaguarina
KJ381241.1|Schinia jaguarina
KJ388767.1|Schinia jaguarina
GU702544.1|Schinia jaguarina
KJ393500.1|Schinia jaguarina
KJ388140.1|Schinia jaguarina
KJ391864.1|Schinia jaguarina
KJ385997.1|Schinia jaguarina
EU768921.1|Heliolonche modicella
KF492642.1|Heliolonche modicella
KF492548.1|Schinia ligeae
KF492547.1|Schinia ligeae
KF492546.1|Schinia ligeae
EU768959.1|Schinia ligeae
KJ657617.1|Schinia petulans
KJ657633.1|Schinia petulans
KJ657615.1|Schinia petulans
KJ657616.1|Schinia petulans
KJ381413.1|Schinia mortua
KJ385888.1|Schinia mortua
KJ393532.1|Schinia mortua
KJ387568.1|Schinia mortua
KF492111.1|Schinia mortua
KF492112.1|Schinia mortua

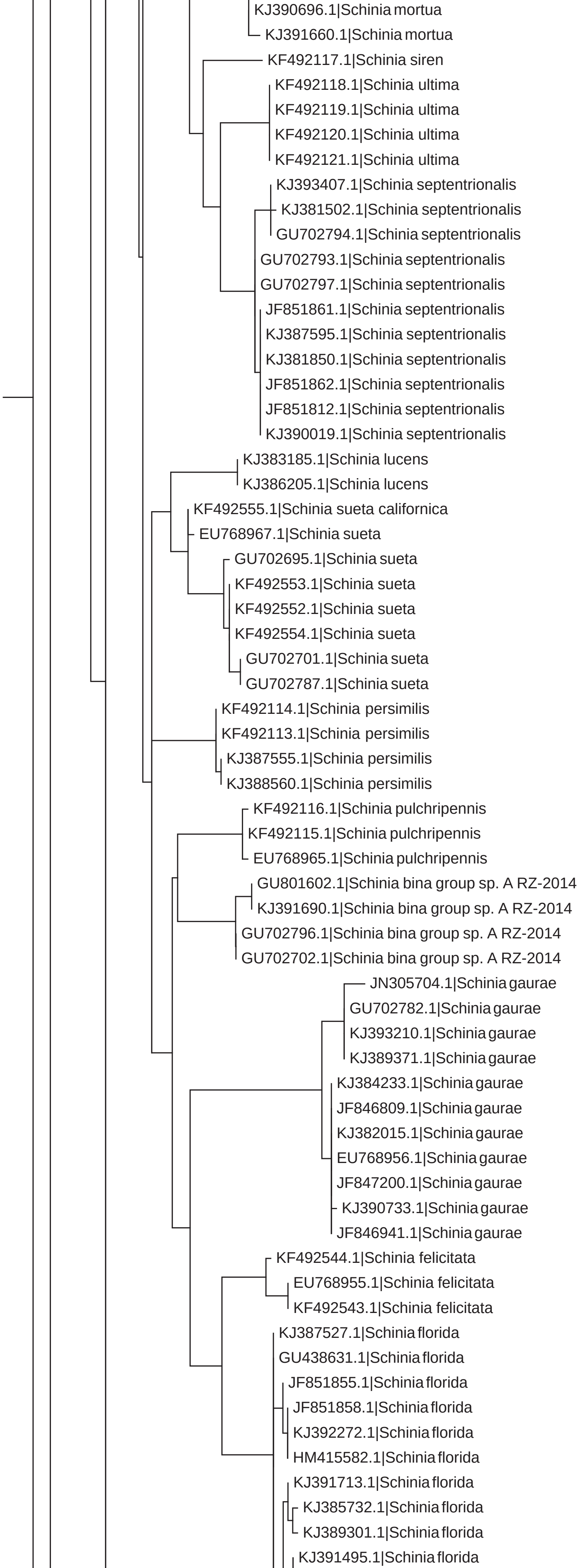
KJ390696.1|Schinia mortua
KJ391660.1|Schinia mortua
KF492117.1|Schinia siren
KF492118.1|Schinia ultima
KF492119.1|Schinia ultima
KF492120.1|Schinia ultima
KF492121.1|Schinia ultima
KJ393407.1|Schinia septentrionalis
KJ381502.1|Schinia septentrionalis
GU702794.1|Schinia septentrionalis
GU702793.1|Schinia septentrionalis
GU702797.1|Schinia septentrionalis
JF851861.1|Schinia septentrionalis
KJ387595.1|Schinia septentrionalis
KJ381850.1|Schinia septentrionalis
JF851862.1|Schinia septentrionalis
JF851812.1|Schinia septentrionalis
KJ390019.1|Schinia septentrionalis
KJ383185.1|Schinia lucens
KJ386205.1|Schinia lucens
KF492555.1|Schinia sueta californica
EU768967.1|Schinia sueta
GU702695.1|Schinia sueta
KF492553.1|Schinia sueta
KF492552.1|Schinia sueta
KF492554.1|Schinia sueta
GU702701.1|Schinia sueta
GU702787.1|Schinia sueta
KF492114.1|Schinia persimilis
KF492113.1|Schinia persimilis
KJ387555.1|Schinia persimilis
KJ388560.1|Schinia persimilis
KF492116.1|Schinia pulchripennis
KF492115.1|Schinia pulchripennis
EU768965.1|Schinia pulchripennis
GU801602.1|Schinia bina group sp. A RZ-2014
KJ391690.1|Schinia bina group sp. A RZ-2014
GU702796.1|Schinia bina group sp. A RZ-2014
GU702702.1|Schinia bina group sp. A RZ-2014
JN305704.1|Schinia gaurae
GU702782.1|Schinia gaurae
KJ393210.1|Schinia gaurae
KJ389371.1|Schinia gaurae
KJ384233.1|Schinia gaurae
JF846809.1|Schinia gaurae
KJ382015.1|Schinia gaurae
EU768956.1|Schinia gaurae
JF847200.1|Schinia gaurae
KJ390733.1|Schinia gaurae
JF846941.1|Schinia gaurae
KF492544.1|Schinia felicitata
EU768955.1|Schinia felicitata
KF492543.1|Schinia felicitata
KJ387527.1|Schinia florida
GU438631.1|Schinia florida
JF851855.1|Schinia florida
JF851858.1|Schinia florida
KJ392272.1|Schinia florida
HM415582.1|Schinia florida
KJ391713.1|Schinia florida
KJ385732.1|Schinia florida
KJ389301.1|Schinia florida
KJ391495.1|Schinia florida

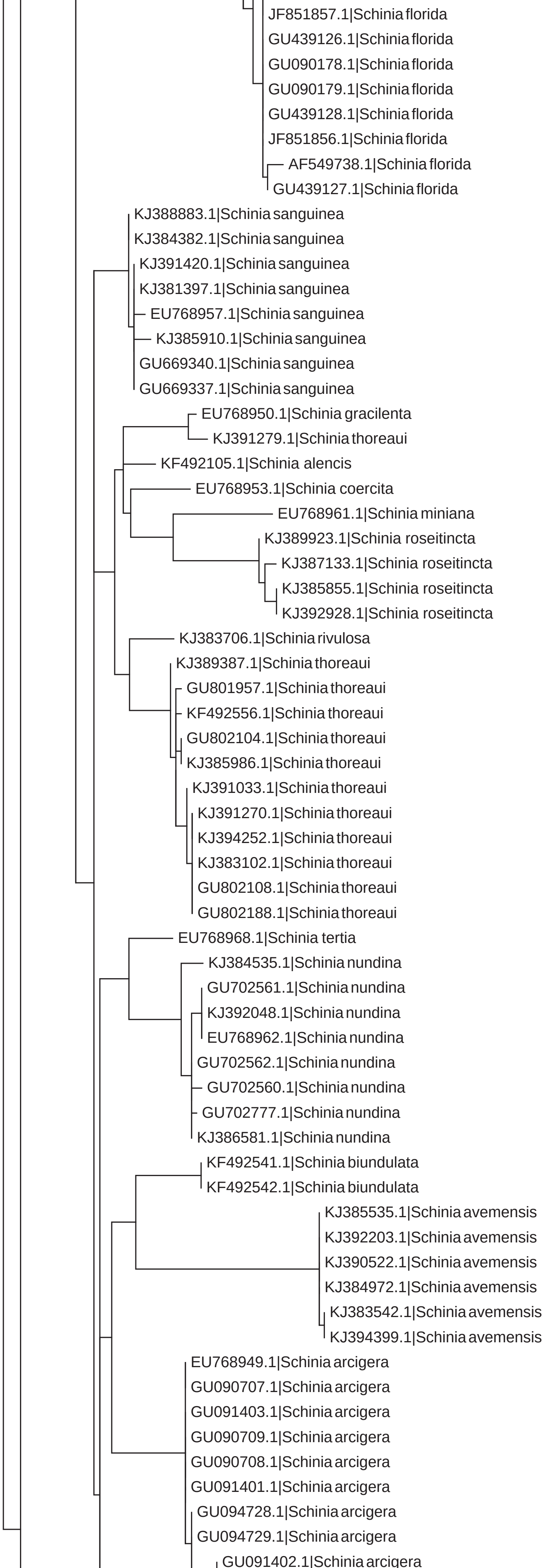
JF851857.1|Schinia florida
GU439126.1|Schinia florida
GU090178.1|Schinia florida
GU090179.1|Schinia florida
GU439128.1|Schinia florida
JF851856.1|Schinia florida
AF549738.1|Schinia florida
GU439127.1|Schinia florida
KJ388883.1|Schinia sanguinea
KJ384382.1|Schinia sanguinea
KJ391420.1|Schinia sanguinea
KJ381397.1|Schinia sanguinea
EU768957.1|Schinia sanguinea
KJ385910.1|Schinia sanguinea
GU669340.1|Schinia sanguinea
GU669337.1|Schinia sanguinea
EU768950.1|Schinia gracilenta
KJ391279.1|Schinia thoreaui
KF492105.1|Schinia alencis
EU768953.1|Schinia coercita
EU768961.1|Schinia miniana
KJ389923.1|Schinia roseitincta
KJ387133.1|Schinia roseitincta
KJ385855.1|Schinia roseitincta
KJ392928.1|Schinia roseitincta
KJ383706.1|Schinia rivulosa
KJ389387.1|Schinia thoreaui
GU801957.1|Schinia thoreaui
KF492556.1|Schinia thoreaui
GU802104.1|Schinia thoreaui
KJ385986.1|Schinia thoreaui
KJ391033.1|Schinia thoreaui
KJ391270.1|Schinia thoreaui
KJ394252.1|Schinia thoreaui
KJ383102.1|Schinia thoreaui
GU802108.1|Schinia thoreaui
GU802188.1|Schinia thoreaui
EU768968.1|Schinia tertia
KJ384535.1|Schinia nundina
GU702561.1|Schinia nundina
KJ392048.1|Schinia nundina
EU768962.1|Schinia nundina
GU702562.1|Schinia nundina
GU702560.1|Schinia nundina
GU702777.1|Schinia nundina
KJ386581.1|Schinia nundina
KF492541.1|Schinia biundulata
KF492542.1|Schinia biundulata
KJ385535.1|Schinia avemensis
KJ392203.1|Schinia avemensis
KJ390522.1|Schinia avemensis
KJ384972.1|Schinia avemensis
KJ383542.1|Schinia avemensis
KJ394399.1|Schinia avemensis
EU768949.1|Schinia arcigera
GU090707.1|Schinia arcigera
GU091403.1|Schinia arcigera
GU090709.1|Schinia arcigera
GU090708.1|Schinia arcigera
GU091401.1|Schinia arcigera
GU094728.1|Schinia arcigera
GU094729.1|Schinia arcigera
GU091402.1|Schinia arcigera

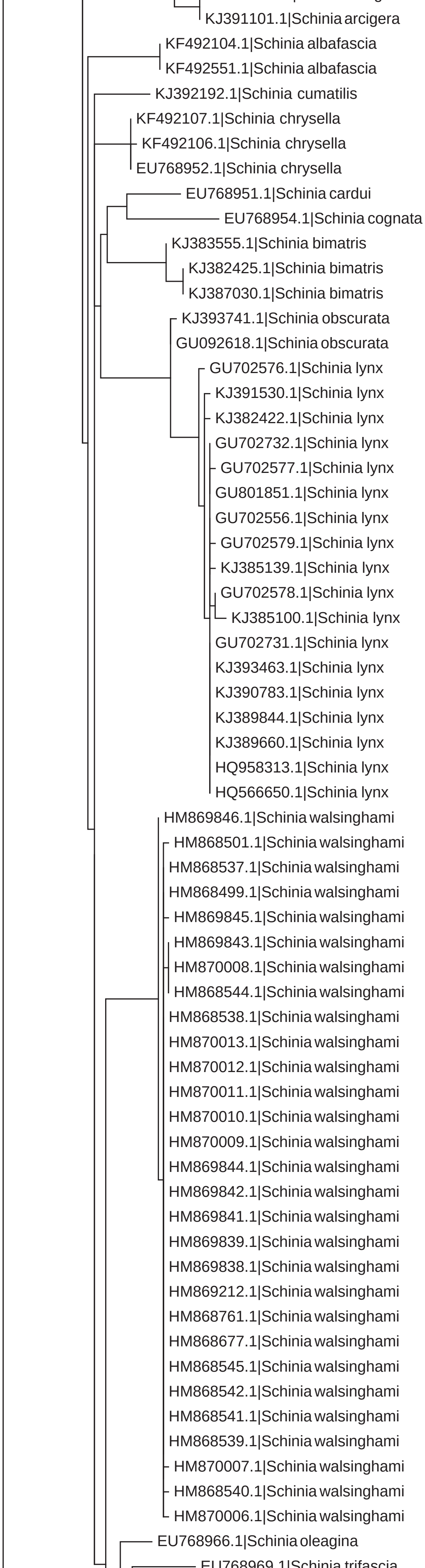
KJ391101.1|Schinia arcigera
KF492104.1|Schinia albafascia
KF492551.1|Schinia albafascia
KJ392192.1|Schinia cumatilis
KF492107.1|Schinia chrysella
KF492106.1|Schinia chrysella
EU768952.1|Schinia chrysella
EU768951.1|Schinia cardui
EU768954.1|Schinia cognata
KJ383555.1|Schinia bimatris
KJ382425.1|Schinia bimatris
KJ387030.1|Schinia bimatris
KJ393741.1|Schinia obscurata
GU092618.1|Schinia obscurata
GU702576.1|Schinia lynx
KJ391530.1|Schinia lynx
KJ382422.1|Schinia lynx
GU702732.1|Schinia lynx
GU702577.1|Schinia lynx
GU801851.1|Schinia lynx
GU702556.1|Schinia lynx
GU702579.1|Schinia lynx
KJ385139.1|Schinia lynx
GU702578.1|Schinia lynx
KJ385100.1|Schinia lynx
GU702731.1|Schinia lynx
KJ393463.1|Schinia lynx
KJ390783.1|Schinia lynx
KJ389844.1|Schinia lynx
KJ389660.1|Schinia lynx
HQ958313.1|Schinia lynx
HQ566650.1|Schinia lynx
HM869846.1|Schinia walsinghami
HM868501.1|Schinia walsinghami
HM868537.1|Schinia walsinghami
HM868499.1|Schinia walsinghami
HM869845.1|Schinia walsinghami
HM869843.1|Schinia walsinghami
HM870008.1|Schinia walsinghami
HM868544.1|Schinia walsinghami
HM868538.1|Schinia walsinghami
HM870013.1|Schinia walsinghami
HM870012.1|Schinia walsinghami
HM870011.1|Schinia walsinghami
HM870010.1|Schinia walsinghami
HM870009.1|Schinia walsinghami
HM869844.1|Schinia walsinghami
HM869842.1|Schinia walsinghami
HM869841.1|Schinia walsinghami
HM869839.1|Schinia walsinghami
HM869838.1|Schinia walsinghami
HM869212.1|Schinia walsinghami
HM868761.1|Schinia walsinghami
HM868677.1|Schinia walsinghami
HM868545.1|Schinia walsinghami
HM868542.1|Schinia walsinghami
HM868541.1|Schinia walsinghami
HM868539.1|Schinia walsinghami
HM870007.1|Schinia walsinghami
HM868540.1|Schinia walsinghami
HM870006.1|Schinia walsinghami
EU768966.1|Schinia oleagina
EU768969.1|Schinia trifascia

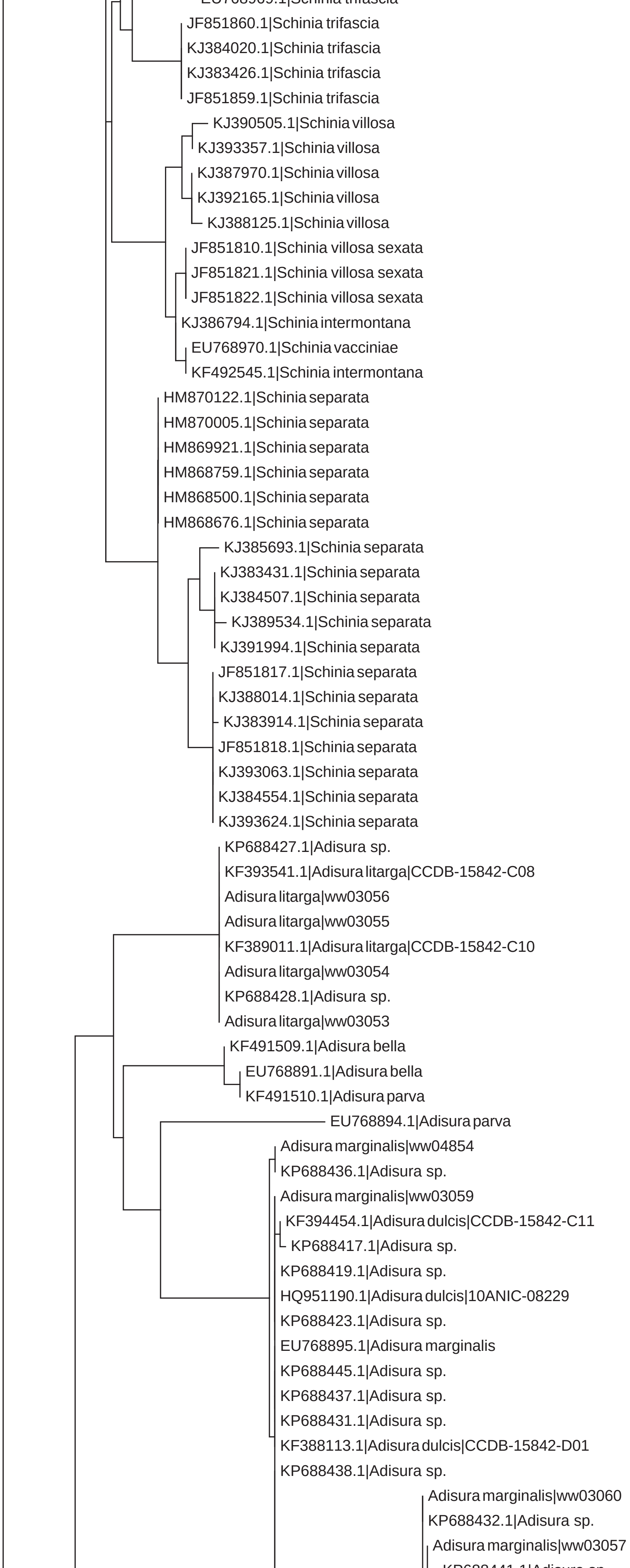
JF851860.1|Schinia trifascia
KJ384020.1|Schinia trifascia
KJ383426.1|Schinia trifascia
JF851859.1|Schinia trifascia
KJ390505.1|Schinia villosa
KJ393357.1|Schinia villosa
KJ387970.1|Schinia villosa
KJ392165.1|Schinia villosa
KJ388125.1|Schinia villosa
JF851810.1|Schinia villosa sexata
JF851821.1|Schinia villosa sexata
JF851822.1|Schinia villosa sexata
KJ386794.1|Schinia intermontana
EU768970.1|Schinia vacciniae
KF492545.1|Schinia intermontana
HM870122.1|Schinia separata
HM870005.1|Schinia separata
HM869921.1|Schinia separata
HM868759.1|Schinia separata
HM868500.1|Schinia separata
HM868676.1|Schinia separata
KJ385693.1|Schinia separata
KJ383431.1|Schinia separata
KJ384507.1|Schinia separata
KJ389534.1|Schinia separata
KJ391994.1|Schinia separata
JF851817.1|Schinia separata
KJ388014.1|Schinia separata
KJ383914.1|Schinia separata
JF851818.1|Schinia separata
KJ393063.1|Schinia separata
KJ384554.1|Schinia separata
KJ393624.1|Schinia separata
KP688427.1|Adisura sp.
KF393541.1|Adisura litarga|CCDB-15842-C08
Adisura litarga|ww03056
Adisura litarga|ww03055
KF389011.1|Adisura litarga|CCDB-15842-C10
Adisura litarga|ww03054
KP688428.1|Adisura sp.
Adisura litarga|ww03053
KF491509.1|Adisura bella
EU768891.1|Adisura bella
KF491510.1|Adisura parva
EU768894.1|Adisura parva
Adisura marginalis|ww04854
KP688436.1|Adisura sp.
Adisura marginalis|ww03059
KF394454.1|Adisura dulcis|CCDB-15842-C11
KP688417.1|Adisura sp.
KP688419.1|Adisura sp.
HQ951190.1|Adisura dulcis|10ANIC-08229
KP688423.1|Adisura sp.
EU768895.1|Adisura marginalis
KP688445.1|Adisura sp.
KP688437.1|Adisura sp.
KP688431.1|Adisura sp.
KF388113.1|Adisura dulcis|CCDB-15842-D01
KP688438.1|Adisura sp.
Adisura marginalis|ww03060
KP688432.1|Adisura sp.
Adisura marginalis|ww03057

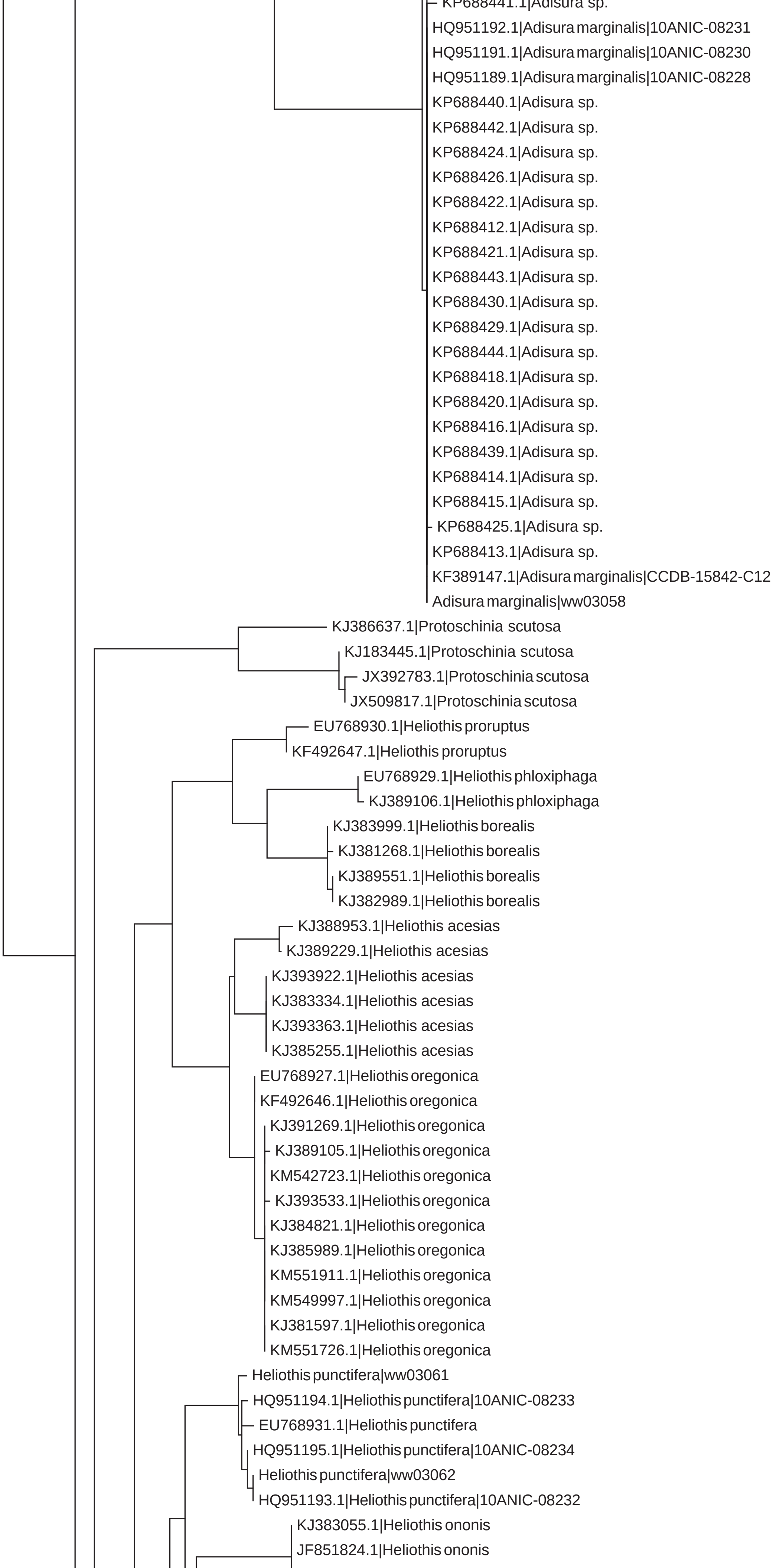

KP688441.1|Adisura sp.
HQ951192.1|Adisura marginalis|10ANIC-08231
HQ951191.1|Adisura marginalis|10ANIC-08230
HQ951189.1|Adisura marginalis|10ANIC-08228
KP688440.1|Adisura sp.
KP688442.1|Adisura sp.
KP688424.1|Adisura sp.
KP688426.1|Adisura sp.
KP688422.1|Adisura sp.
KP688412.1|Adisura sp.
KP688421.1|Adisura sp.
KP688443.1|Adisura sp.
KP688430.1|Adisura sp.
KP688429.1|Adisura sp.
KP688444.1|Adisura sp.
KP688418.1|Adisura sp.
KP688420.1|Adisura sp.
KP688416.1|Adisura sp.
KP688439.1|Adisura sp.
KP688414.1|Adisura sp.
KP688415.1|Adisura sp.
KP688425.1|Adisura sp.
KP688413.1|Adisura sp.
KF389147.1|Adisura marginalis|CCDB-15842-C12
Adisura marginalis|ww03058
KJ386637.1|Protoschinia scutosa
KJ183445.1|Protoschinia scutosa
JX392783.1|Protoschinia scutosa
JX509817.1|Protoschinia scutosa
EU768930.1|Heliothis proruptus
KF492647.1|Heliothis proruptus
EU768929.1|Heliothis phloxiphaga
KJ389106.1|Heliothis phloxiphaga
KJ383999.1|Heliothis borealis
KJ381268.1|Heliothis borealis
KJ389551.1|Heliothis borealis
KJ382989.1|Heliothis borealis
KJ388953.1|Heliothis acesias
KJ389229.1|Heliothis acesias
KJ393922.1|Heliothis acesias
KJ383334.1|Heliothis acesias
KJ393363.1|Heliothis acesias
KJ385255.1|Heliothis acesias
EU768927.1|Heliothis oregonica
KF492646.1|Heliothis oregonica
KJ391269.1|Heliothis oregonica
KJ389105.1|Heliothis oregonica
KM542723.1|Heliothis oregonica
KJ393533.1|Heliothis oregonica
KJ384821.1|Heliothis oregonica
KJ385989.1|Heliothis oregonica
KM551911.1|Heliothis oregonica
KM549997.1|Heliothis oregonica
KJ381597.1|Heliothis oregonica
KM551726.1|Heliothis oregonica
Heliothis punctifera|ww03061
HQ951194.1|Heliothis punctifera|10ANIC-08233
EU768931.1|Heliothis punctifera
HQ951195.1|Heliothis punctifera|10ANIC-08234
Heliothis punctifera|ww03062
HQ951193.1|Heliothis punctifera|10ANIC-08232
KJ383055.1|Heliothis ononis
JF851824.1|Heliothis ononis

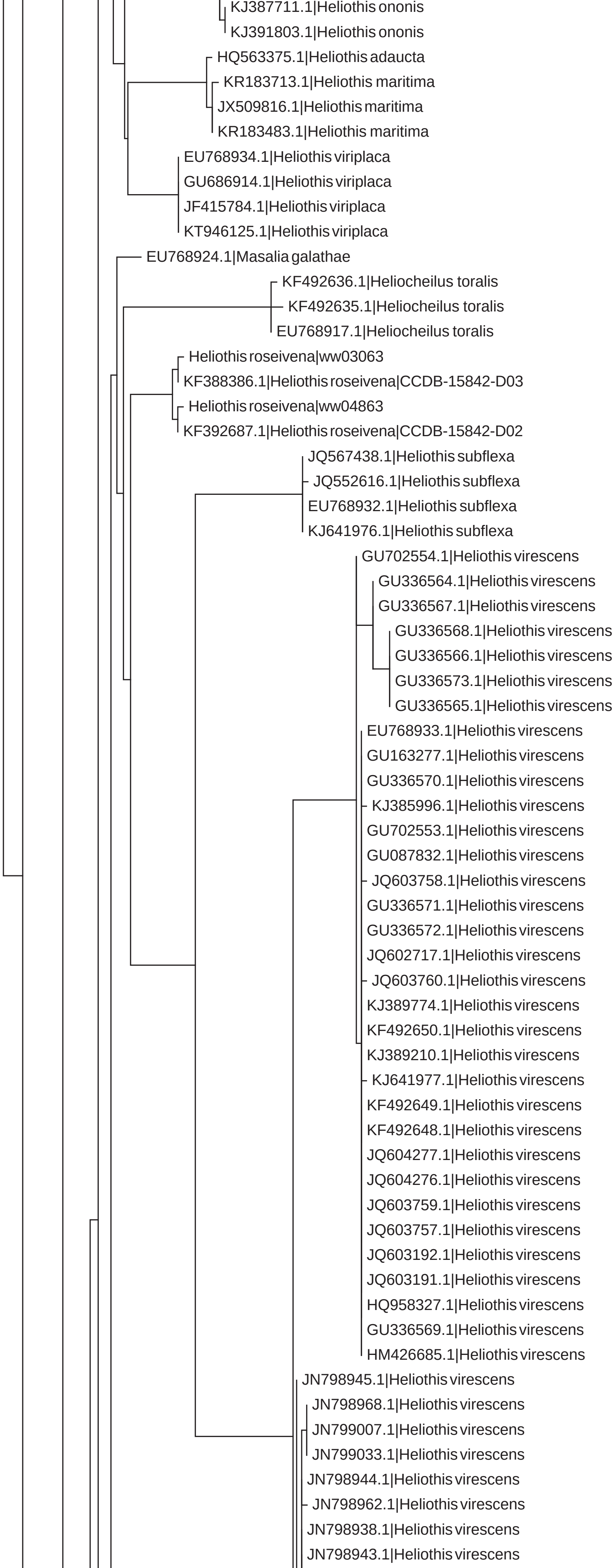
KJ387711.1|Heliothis ononis
KJ391803.1|Heliothis ononis
HQ563375.1|Heliothis adaucta
KR183713.1|Heliothis maritima
JX509816.1|Heliothis maritima
KR183483.1|Heliothis maritima
EU768934.1|Heliothis viriplaca
GU686914.1|Heliothis viriplaca
JF415784.1|Heliothis viriplaca
KT946125.1|Heliothis viriplaca
EU768924.1|Masalia galathae
KF492636.1|Heliocheilus toralis
KF492635.1|Heliocheilus toralis
EU768917.1|Heliocheilus toralis
Heliothis roseivena|ww03063
KF388386.1|Heliothis roseivena|CCDB-15842-D03
Heliothis roseivena|ww04863
KF392687.1|Heliothis roseivena|CCDB-15842-D02
JQ567438.1|Heliothis subflexa
JQ552616.1|Heliothis subflexa
EU768932.1|Heliothis subflexa
KJ641976.1|Heliothis subflexa
GU702554.1|Heliothis virescens
GU336564.1|Heliothis virescens
GU336567.1|Heliothis virescens
GU336568.1|Heliothis virescens
GU336566.1|Heliothis virescens
GU336573.1|Heliothis virescens
GU336565.1|Heliothis virescens
EU768933.1|Heliothis virescens
GU163277.1|Heliothis virescens
GU336570.1|Heliothis virescens
KJ385996.1|Heliothis virescens
GU702553.1|Heliothis virescens
GU087832.1|Heliothis virescens
JQ603758.1|Heliothis virescens
GU336571.1|Heliothis virescens
GU336572.1|Heliothis virescens
JQ602717.1|Heliothis virescens
JQ603760.1|Heliothis virescens
KJ389774.1|Heliothis virescens
KF492650.1|Heliothis virescens
KJ389210.1|Heliothis virescens
KJ641977.1|Heliothis virescens
KF492649.1|Heliothis virescens
KF492648.1|Heliothis virescens
JQ604277.1|Heliothis virescens
JQ604276.1|Heliothis virescens
JQ603759.1|Heliothis virescens
JQ603757.1|Heliothis virescens
JQ603192.1|Heliothis virescens
JQ603191.1|Heliothis virescens
HQ958327.1|Heliothis virescens
GU336569.1|Heliothis virescens
HM426685.1|Heliothis virescens
JN798945.1|Heliothis virescens
JN798968.1|Heliothis virescens
JN799007.1|Heliothis virescens
JN799033.1|Heliothis virescens
JN798944.1|Heliothis virescens
JN798962.1|Heliothis virescens
JN798938.1|Heliothis virescens
JN798943.1|Heliothis virescens

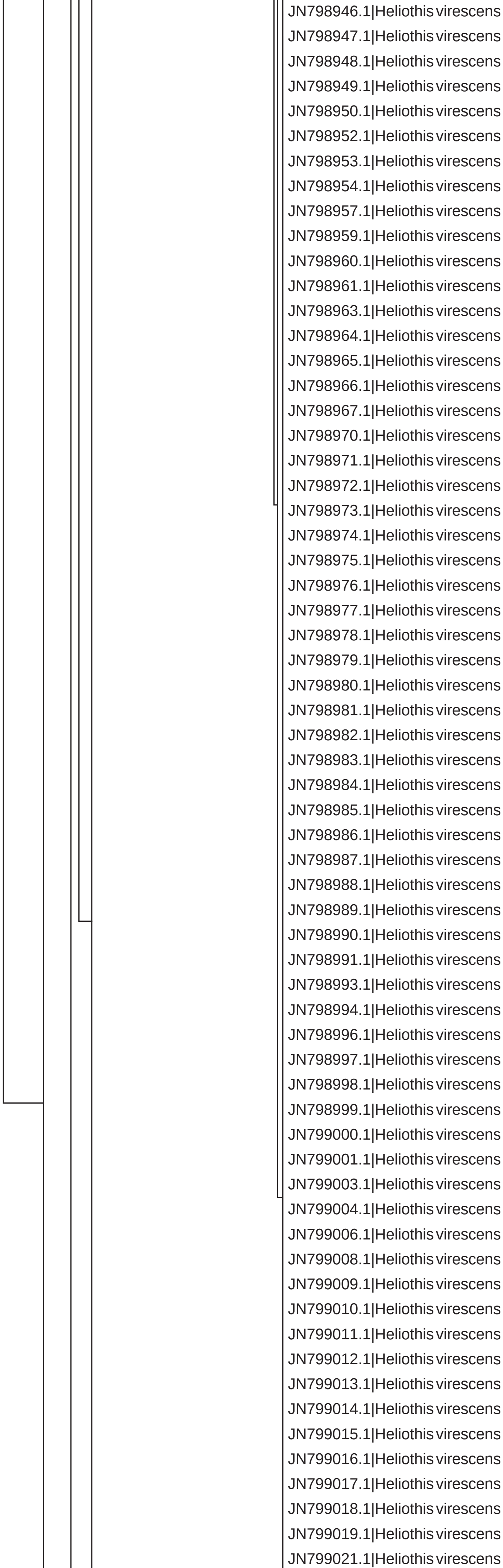

JN798946.1|Heliothis virescens
JN798947.1|Heliothis virescens
JN798948.1|Heliothis virescens
JN798949.1|Heliothis virescens
JN798950.1|Heliothis virescens
JN798952.1|Heliothis virescens
JN798953.1|Heliothis virescens
JN798954.1|Heliothis virescens
JN798957.1|Heliothis virescens
JN798959.1|Heliothis virescens
JN798960.1|Heliothis virescens
JN798961.1|Heliothis virescens
JN798963.1|Heliothis virescens
JN798964.1|Heliothis virescens
JN798965.1|Heliothis virescens
JN798966.1|Heliothis virescens
JN798967.1|Heliothis virescens
JN798970.1|Heliothis virescens
JN798971.1|Heliothis virescens
JN798972.1|Heliothis virescens
JN798973.1|Heliothis virescens
JN798974.1|Heliothis virescens
JN798975.1|Heliothis virescens
JN798976.1|Heliothis virescens
JN798977.1|Heliothis virescens
JN798978.1|Heliothis virescens
JN798979.1|Heliothis virescens
JN798980.1|Heliothis virescens
JN798981.1|Heliothis virescens
JN798982.1|Heliothis virescens
JN798983.1|Heliothis virescens
JN798984.1|Heliothis virescens
JN798985.1|Heliothis virescens
JN798986.1|Heliothis virescens
JN798987.1|Heliothis virescens
JN798988.1|Heliothis virescens
JN798989.1|Heliothis virescens
JN798990.1|Heliothis virescens
JN798991.1|Heliothis virescens
JN798993.1|Heliothis virescens
JN798994.1|Heliothis virescens
JN798996.1|Heliothis virescens
JN798997.1|Heliothis virescens
JN798998.1|Heliothis virescens
JN798999.1|Heliothis virescens
JN799000.1|Heliothis virescens
JN799001.1|Heliothis virescens
JN799003.1|Heliothis virescens
JN799004.1|Heliothis virescens
JN799006.1|Heliothis virescens
JN799008.1|Heliothis virescens
JN799009.1|Heliothis virescens
JN799010.1|Heliothis virescens
JN799011.1|Heliothis virescens
JN799012.1|Heliothis virescens
JN799013.1|Heliothis virescens
JN799014.1|Heliothis virescens
JN799015.1|Heliothis virescens
JN799016.1|Heliothis virescens
JN799017.1|Heliothis virescens
JN799018.1|Heliothis virescens
JN799019.1|Heliothis virescens
JN799021.1|Heliothis virescens

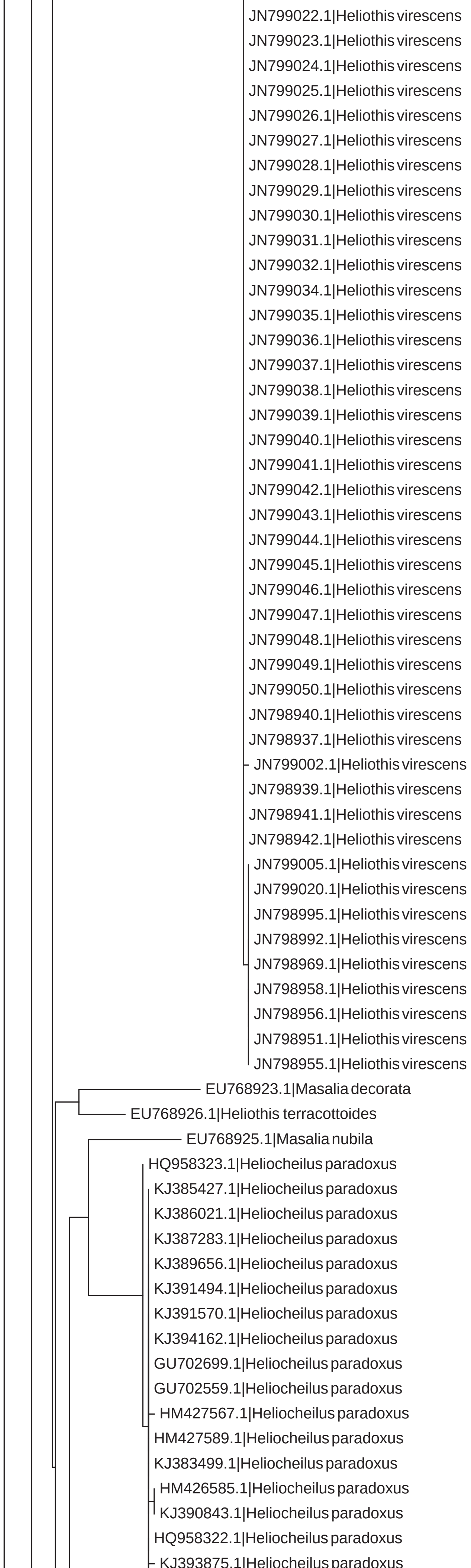

JN799022.1|Heliothis virescens
JN799023.1|Heliothis virescens
JN799024.1|Heliothis virescens
JN799025.1|Heliothis virescens
JN799026.1|Heliothis virescens
JN799027.1|Heliothis virescens
JN799028.1|Heliothis virescens
JN799029.1|Heliothis virescens
JN799030.1|Heliothis virescens
JN799031.1|Heliothis virescens
JN799032.1|Heliothis virescens
JN799034.1|Heliothis virescens
JN799035.1|Heliothis virescens
JN799036.1|Heliothis virescens
JN799037.1|Heliothis virescens
JN799038.1|Heliothis virescens
JN799039.1|Heliothis virescens
JN799040.1|Heliothis virescens
JN799041.1|Heliothis virescens
JN799042.1|Heliothis virescens
JN799043.1|Heliothis virescens
JN799044.1|Heliothis virescens
JN799045.1|Heliothis virescens
JN799046.1|Heliothis virescens
JN799047.1|Heliothis virescens
JN799048.1|Heliothis virescens
JN799049.1|Heliothis virescens
JN799050.1|Heliothis virescens
JN798940.1|Heliothis virescens
JN798937.1|Heliothis virescens
JN799002.1|Heliothis virescens
JN798939.1|Heliothis virescens
JN798941.1|Heliothis virescens
JN798942.1|Heliothis virescens
JN799005.1|Heliothis virescens
JN799020.1|Heliothis virescens
JN798995.1|Heliothis virescens
JN798992.1|Heliothis virescens
JN798969.1|Heliothis virescens
JN798958.1|Heliothis virescens
JN798956.1|Heliothis virescens
JN798951.1|Heliothis virescens
JN798955.1|Heliothis virescens
EU768923.1|Masalia decorata
EU768926.1|Heliothis terracottoides
EU768925.1|Masalia nubila
HQ958323.1|Heliocheilus paradoxus
KJ385427.1|Heliocheilus paradoxus
KJ386021.1|Heliocheilus paradoxus
KJ387283.1|Heliocheilus paradoxus
KJ389656.1|Heliocheilus paradoxus
KJ391494.1|Heliocheilus paradoxus
KJ391570.1|Heliocheilus paradoxus
KJ394162.1|Heliocheilus paradoxus
GU702699.1|Heliocheilus paradoxus
GU702559.1|Heliocheilus paradoxus
HM427567.1|Heliocheilus paradoxus
HM427589.1|Heliocheilus paradoxus
KJ383499.1|Heliocheilus paradoxus
HM426585.1|Heliocheilus paradoxus
KJ390843.1|Heliocheilus paradoxus
HQ958322.1|Heliocheilus paradoxus
KJ393875.1|Heliocheilus paradoxus

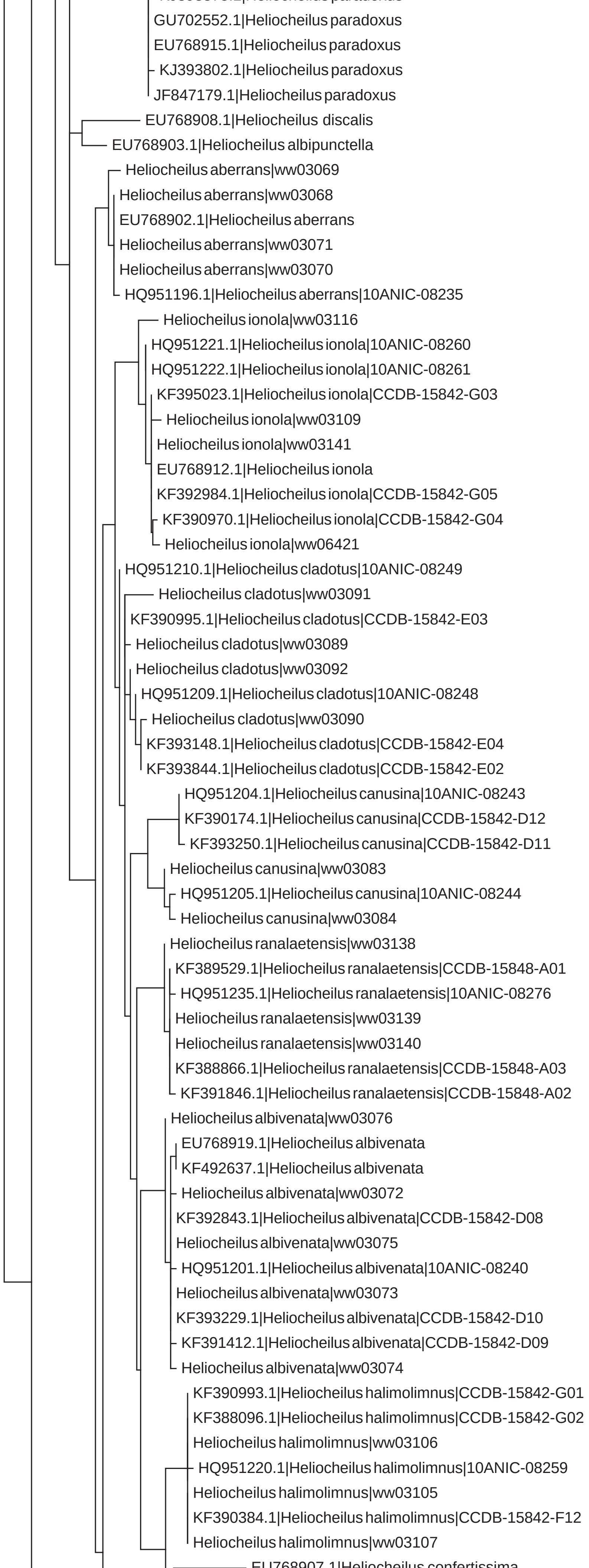
GU702552.1|Heliocheilus paradoxus
EU768915.1|Heliocheilus paradoxus
KJ393802.1|Heliocheilus paradoxus
JF847179.1|Heliocheilus paradoxus
EU768908.1|Heliocheilus discalis
EU768903.1|Heliocheilus albipunctella
Heliocheilus aberrans|ww03069
Heliocheilus aberrans|ww03068
EU768902.1|Heliocheilus aberrans
Heliocheilus aberrans|ww03071
Heliocheilus aberrans|ww03070
HQ951196.1|Heliocheilus aberrans|10ANIC-08235
Heliocheilus ionola|ww03116
HQ951221.1|Heliocheilus ionola|10ANIC-08260
HQ951222.1|Heliocheilus ionola|10ANIC-08261
KF395023.1|Heliocheilus ionola|CCDB-15842-G03
Heliocheilus ionola|ww03109
Heliocheilus ionola|ww03141
EU768912.1|Heliocheilus ionola
KF392984.1|Heliocheilus ionola|CCDB-15842-G05
KF390970.1|Heliocheilus ionola|CCDB-15842-G04
Heliocheilus ionola|ww06421
HQ951210.1|Heliocheilus cladotus|10ANIC-08249
Heliocheilus cladotus|ww03091
KF390995.1|Heliocheilus cladotus|CCDB-15842-E03
Heliocheilus cladotus|ww03089
Heliocheilus cladotus|ww03092
HQ951209.1|Heliocheilus cladotus|10ANIC-08248
Heliocheilus cladotus|ww03090
KF393148.1|Heliocheilus cladotus|CCDB-15842-E04
KF393844.1|Heliocheilus cladotus|CCDB-15842-E02
HQ951204.1|Heliocheilus canusina|10ANIC-08243
KF390174.1|Heliocheilus canusina|CCDB-15842-D12
KF393250.1|Heliocheilus canusina|CCDB-15842-D11
Heliocheilus canusina|ww03083
HQ951205.1|Heliocheilus canusina|10ANIC-08244
Heliocheilus canusina|ww03084
Heliocheilus ranalaetensis|ww03138
KF389529.1|Heliocheilus ranalaetensis|CCDB-15848-A01
HQ951235.1|Heliocheilus ranalaetensis|10ANIC-08276
Heliocheilus ranalaetensis|ww03139
Heliocheilus ranalaetensis|ww03140
KF388866.1|Heliocheilus ranalaetensis|CCDB-15848-A03
KF391846.1|Heliocheilus ranalaetensis|CCDB-15848-A02
Heliocheilus albivenata|ww03076
EU768919.1|Heliocheilus albivenata
KF492637.1|Heliocheilus albivenata
Heliocheilus albivenata|ww03072
KF392843.1|Heliocheilus albivenata|CCDB-15842-D08
Heliocheilus albivenata|ww03075
HQ951201.1|Heliocheilus albivenata|10ANIC-08240
Heliocheilus albivenata|ww03073
KF393229.1|Heliocheilus albivenata|CCDB-15842-D10
KF391412.1|Heliocheilus albivenata|CCDB-15842-D09
Heliocheilus albivenata|ww03074
KF390993.1|Heliocheilus halimolimnus|CCDB-15842-G01
KF388096.1|Heliocheilus halimolimnus|CCDB-15842-G02
Heliocheilus halimolimnus|ww03106
HQ951220.1|Heliocheilus halimolimnus|10ANIC-08259
Heliocheilus halimolimnus|ww03105
KF390384.1|Heliocheilus halimolimnus|CCDB-15842-F12
Heliocheilus halimolimnus|ww03107

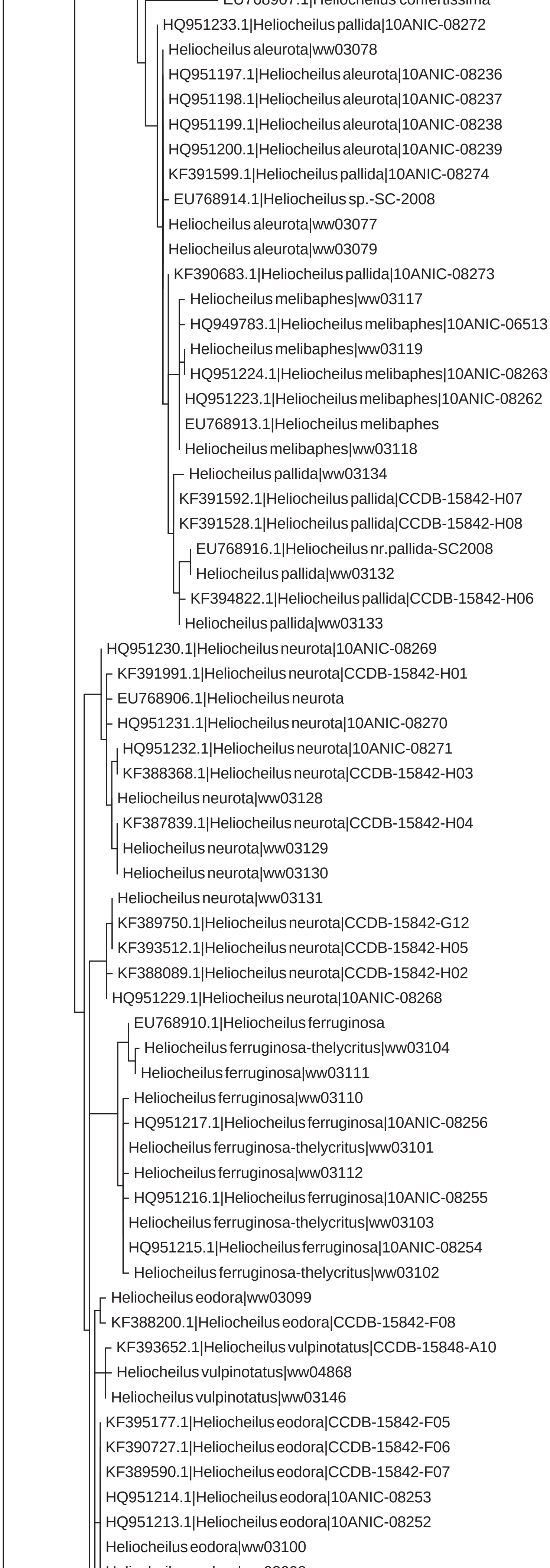
EU768907.1|Heliocheilus confertissima
HQ951233.1|Heliocheilus pallida|10ANIC-08272
Heliocheilus aleurota|ww03078
HQ951197.1|Heliocheilus aleurota|10ANIC-08236
HQ951198.1|Heliocheilus aleurota|10ANIC-08237
HQ951199.1|Heliocheilus aleurota|10ANIC-08238
HQ951200.1|Heliocheilus aleurota|10ANIC-08239
KF391599.1|Heliocheilus pallida|10ANIC-08274
EU768914.1|Heliocheilus sp.-SC-2008
Heliocheilus aleurota|ww03077
Heliocheilus aleurota|ww03079
KF390683.1|Heliocheilus pallida|10ANIC-08273
Heliocheilus melibaphes|ww03117
HQ949783.1|Heliocheilus melibaphes|10ANIC-06513
Heliocheilus melibaphes|ww03119
HQ951224.1|Heliocheilus melibaphes|10ANIC-08263
HQ951223.1|Heliocheilus melibaphes|10ANIC-08262
EU768913.1|Heliocheilus melibaphes
Heliocheilus melibaphes|ww03118
Heliocheilus pallida|ww03134
KF391592.1|Heliocheilus pallida|CCDB-15842-H07
KF391528.1|Heliocheilus pallida|CCDB-15842-H08
EU768916.1|Heliocheilus nr.pallida-SC2008
Heliocheilus pallida|ww03132
KF394822.1|Heliocheilus pallida|CCDB-15842-H06
Heliocheilus pallida|ww03133
HQ951230.1|Heliocheilus neurota|10ANIC-08269
KF391991.1|Heliocheilus neurota|CCDB-15842-H01
EU768906.1|Heliocheilus neurota
HQ951231.1|Heliocheilus neurota|10ANIC-08270
HQ951232.1|Heliocheilus neurota|10ANIC-08271
KF388368.1|Heliocheilus neurota|CCDB-15842-H03
Heliocheilus neurota|ww03128
KF387839.1|Heliocheilus neurota|CCDB-15842-H04
Heliocheilus neurota|ww03129
Heliocheilus neurota|ww03130
Heliocheilus neurota|ww03131
KF389750.1|Heliocheilus neurota|CCDB-15842-G12
KF393512.1|Heliocheilus neurota|CCDB-15842-H05
KF388089.1|Heliocheilus neurota|CCDB-15842-H02
HQ951229.1|Heliocheilus neurota|10ANIC-08268
EU768910.1|Heliocheilus ferruginosa
Heliocheilus ferruginosa-thelycritus|ww03104
Heliocheilus ferruginosa|ww03111
Heliocheilus ferruginosa|ww03110
HQ951217.1|Heliocheilus ferruginosa|10ANIC-08256
Heliocheilus ferruginosa-thelycritus|ww03101
Heliocheilus ferruginosa|ww03112
HQ951216.1|Heliocheilus ferruginosa|10ANIC-08255
Heliocheilus ferruginosa-thelycritus|ww03103
HQ951215.1|Heliocheilus ferruginosa|10ANIC-08254
Heliocheilus ferruginosa-thelycritus|ww03102
Heliocheilus eodora|ww03099
KF388200.1|Heliocheilus eodora|CCDB-15842-F08
KF393652.1|Heliocheilus vulpinotatus|CCDB-15848-A10
Heliocheilus vulpinotatus|ww04868
Heliocheilus vulpinotatus|ww03146
KF395177.1|Heliocheilus eodora|CCDB-15842-F05
KF390727.1|Heliocheilus eodora|CCDB-15842-F06
KF389590.1|Heliocheilus eodora|CCDB-15842-F07
HQ951214.1|Heliocheilus eodora|10ANIC-08253
HQ951213.1|Heliocheilus eodora|10ANIC-08252
Heliocheilus eodora|ww03100

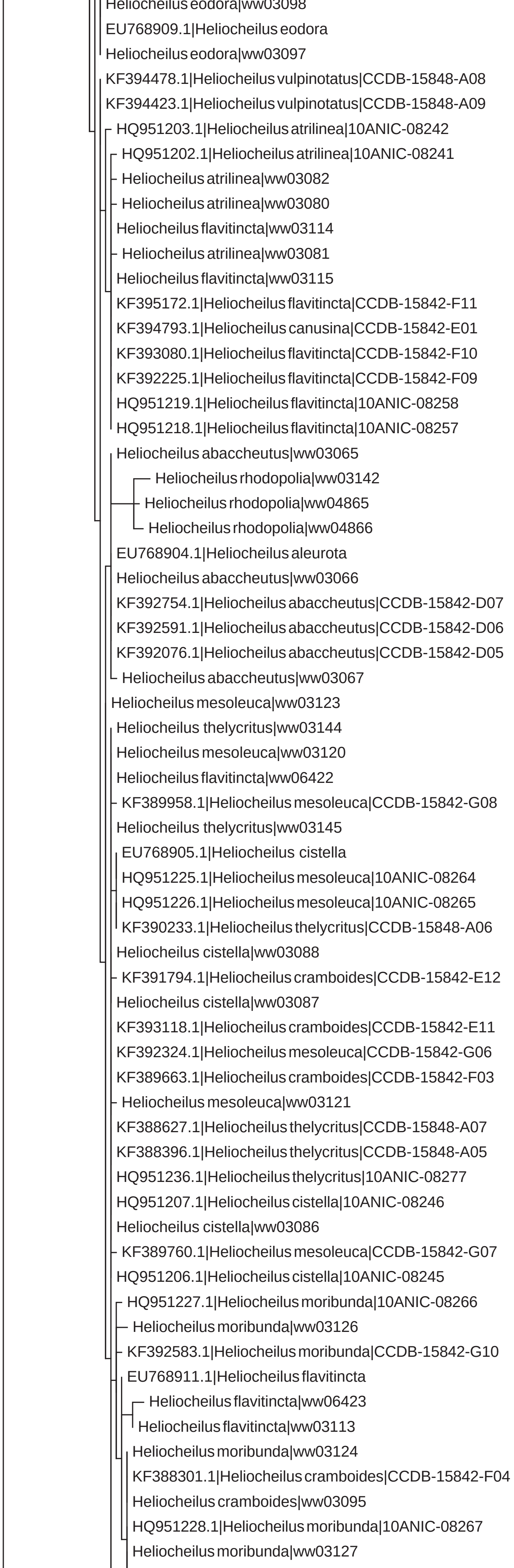

Heliocheilus eodora|ww03098
EU768909.1|Heliocheilus eodora
Heliocheilus eodora|ww03097
KF394478.1|Heliocheilus vulpinotatus|CCDB-15848-A08
KF394423.1|Heliocheilus vulpinotatus|CCDB-15848-A09
HQ951203.1|Heliocheilus atrilinea|10ANIC-08242
HQ951202.1|Heliocheilus atrilinea|10ANIC-08241
Heliocheilus atrilinea|ww03082
Heliocheilus atrilinea|ww03080
Heliocheilus flavitincta|ww03114
Heliocheilus atrilinea|ww03081
Heliocheilus flavitincta|ww03115
KF395172.1|Heliocheilus flavitincta|CCDB-15842-F11
KF394793.1|Heliocheilus canusina|CCDB-15842-E01
KF393080.1|Heliocheilus flavitincta|CCDB-15842-F10
KF392225.1|Heliocheilus flavitincta|CCDB-15842-F09
HQ951219.1|Heliocheilus flavitincta|10ANIC-08258
HQ951218.1|Heliocheilus flavitincta|10ANIC-08257
Heliocheilus abaccheutus|ww03065
Heliocheilus rhodopolia|ww03142
Heliocheilus rhodopolia|ww04865
Heliocheilus rhodopolia|ww04866
EU768904.1|Heliocheilus aleurota
Heliocheilus abaccheutus|ww03066
KF392754.1|Heliocheilus abaccheutus|CCDB-15842-D07
KF392591.1|Heliocheilus abaccheutus|CCDB-15842-D06
KF392076.1|Heliocheilus abaccheutus|CCDB-15842-D05
Heliocheilus abaccheutus|ww03067
Heliocheilus mesoleuca|ww03123
Heliocheilus thelycritus|ww03144
Heliocheilus mesoleuca|ww03120
Heliocheilus flavitincta|ww06422
KF389958.1|Heliocheilus mesoleuca|CCDB-15842-G08
Heliocheilus thelycritus|ww03145
EU768905.1|Heliocheilus cistella
HQ951225.1|Heliocheilus mesoleuca|10ANIC-08264
HQ951226.1|Heliocheilus mesoleuca|10ANIC-08265
KF390233.1|Heliocheilus thelycritus|CCDB-15848-A06
Heliocheilus cistella|ww03088
KF391794.1|Heliocheilus cramboides|CCDB-15842-E12
Heliocheilus cistella|ww03087
KF393118.1|Heliocheilus cramboides|CCDB-15842-E11
KF392324.1|Heliocheilus mesoleuca|CCDB-15842-G06
KF389663.1|Heliocheilus cramboides|CCDB-15842-F03
Heliocheilus mesoleuca|ww03121
KF388627.1|Heliocheilus thelycritus|CCDB-15848-A07
KF388396.1|Heliocheilus thelycritus|CCDB-15848-A05
HQ951236.1|Heliocheilus thelycritus|10ANIC-08277
HQ951207.1|Heliocheilus cistella|10ANIC-08246
Heliocheilus cistella|ww03086
KF389760.1|Heliocheilus mesoleuca|CCDB-15842-G07
HQ951206.1|Heliocheilus cistella|10ANIC-08245
HQ951227.1|Heliocheilus moribunda|10ANIC-08266
Heliocheilus moribunda|ww03126
KF392583.1|Heliocheilus moribunda|CCDB-15842-G10
EU768911.1|Heliocheilus flavitincta
Heliocheilus flavitincta|ww06423
Heliocheilus flavitincta|ww03113
Heliocheilus moribunda|ww03124
KF388301.1|Heliocheilus cramboides|CCDB-15842-F04
Heliocheilus cramboides|ww03095
HQ951228.1|Heliocheilus moribunda|10ANIC-08267
Heliocheilus moribunda|ww03127

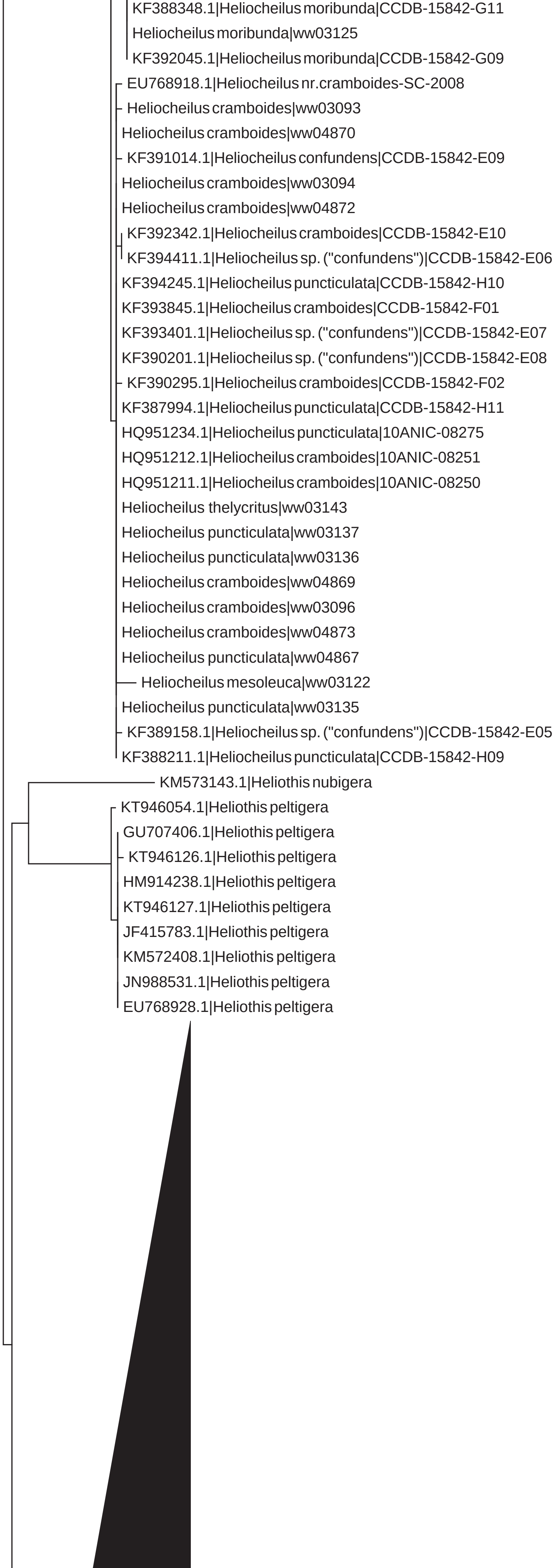
KF388348.1|Heliocheilus moribunda|CCDB-15842-G11
Heliocheilus moribunda|ww03125
KF392045.1|Heliocheilus moribunda|CCDB-15842-G09
EU768918.1|Heliocheilus nr.cramboides-SC-2008
Heliocheilus cramboides|ww03093
Heliocheilus cramboides|ww04870
KF391014.1|Heliocheilus confundens|CCDB-15842-E09
Heliocheilus cramboides|ww03094
Heliocheilus cramboides|ww04872
KF392342.1|Heliocheilus cramboides|CCDB-15842-E10
KF394411.1|Heliocheilus sp. ("confundens")|CCDB-15842-E06
KF394245.1|Heliocheilus puncticulata|CCDB-15842-H10
KF393845.1|Heliocheilus cramboides|CCDB-15842-F01
KF393401.1|Heliocheilus sp. ("confundens")|CCDB-15842-E07
KF390201.1|Heliocheilus sp. ("confundens")|CCDB-15842-E08
KF390295.1|Heliocheilus cramboides|CCDB-15842-F02
KF387994.1|Heliocheilus puncticulata|CCDB-15842-H11
HQ951234.1|Heliocheilus puncticulata|10ANIC-08275
HQ951212.1|Heliocheilus cramboides|10ANIC-08251
HQ951211.1|Heliocheilus cramboides|10ANIC-08250
Heliocheilus thelycritus|ww03143
Heliocheilus puncticulata|ww03137
Heliocheilus puncticulata|ww03136
Heliocheilus cramboides|ww04869
Heliocheilus cramboides|ww03096
Heliocheilus cramboides|ww04873
Heliocheilus puncticulata|ww04867
Heliocheilus mesoleuca|ww03122
Heliocheilus puncticulata|ww03135
KF389158.1|Heliocheilus sp. ("confundens")|CCDB-15842-E05
KF388211.1|Heliocheilus puncticulata|CCDB-15842-H09
KM573143.1|Heliothis nubigera
KT946054.1|Heliothis peltigera
GU707406.1|Heliothis peltigera
KT946126.1|Heliothis peltigera
HM914238.1|Heliothis peltigera
KT946127.1|Heliothis peltigera
JF415783.1|Heliothis peltigera
KM572408.1|Heliothis peltigera
JN988531.1|Heliothis peltigera
EU768928.1|Heliothis peltigera

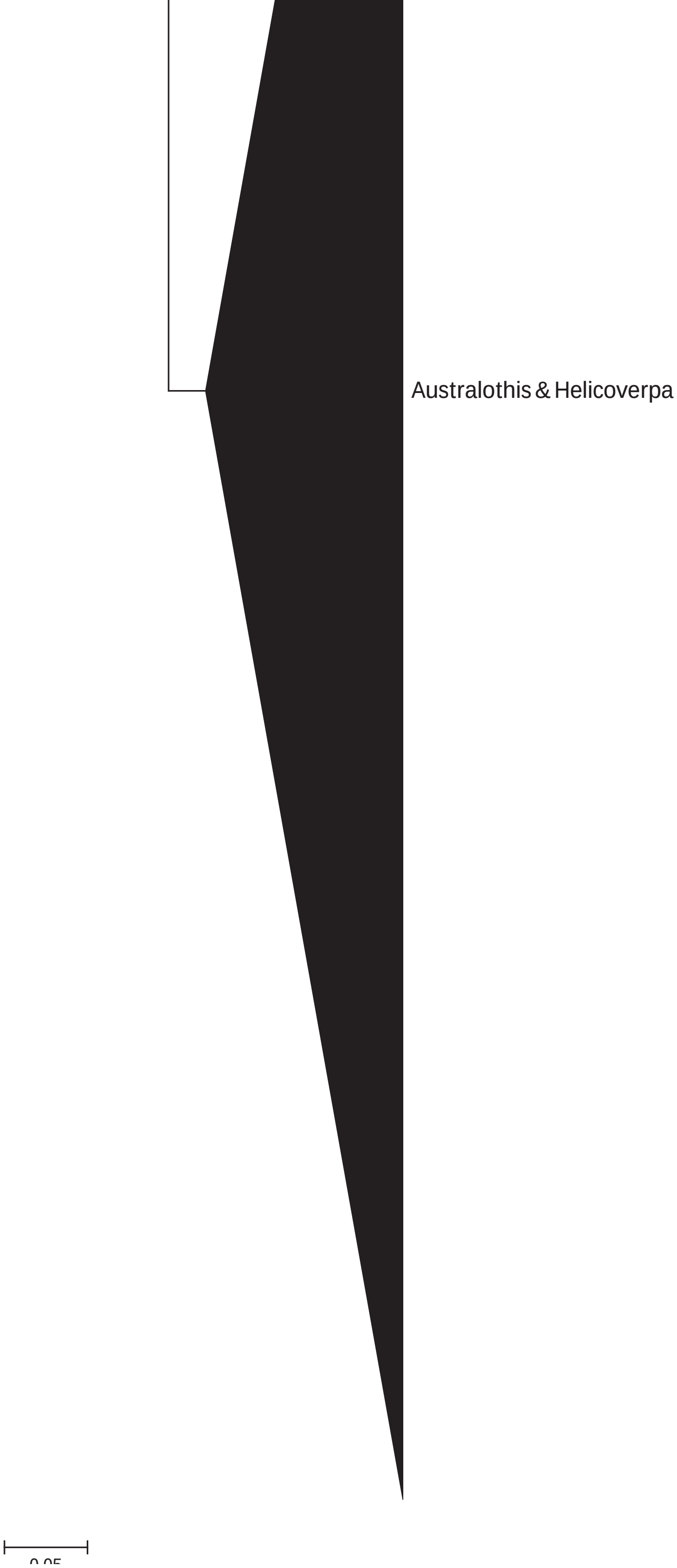
Australothis & Helicoverpa
0.05
