# Supplementary material for: DNA Barcoding the Heliothinae (Lepidoptera: Noctuidae) of Australia and Utility of DNA Barcodes for Pest Identification in Helicoverpa and Relatives
Source: PLoS One. 2016 Aug 10;11(8):e0160895. doi: 10.1371/journal.pone.0160895 (PMC4980029; doi:10.1371/journal.pone.0160895)
Supplement: S2 Fig — (PDF) [file pone.0160895.s002.pdf]

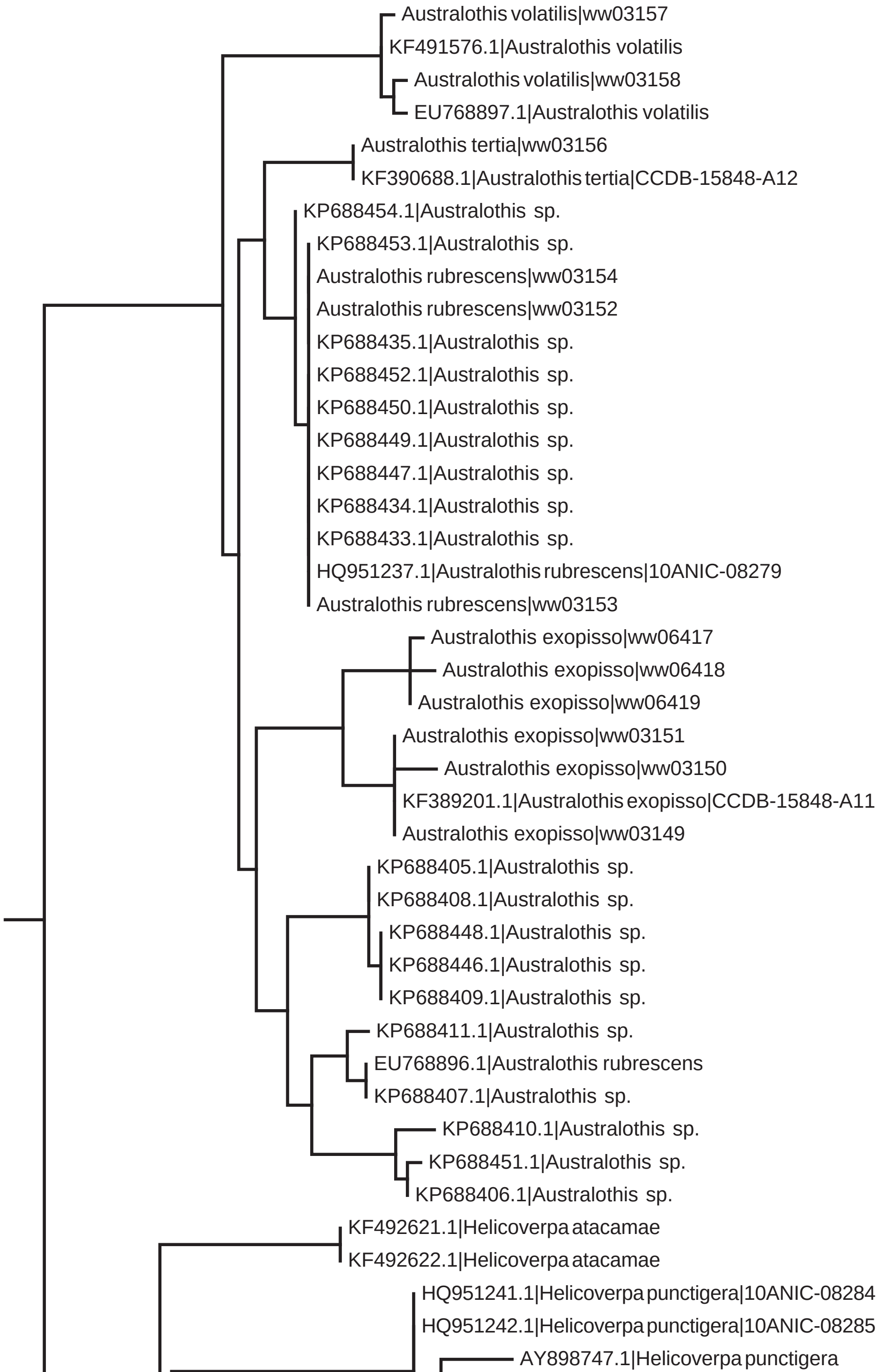
Australothis volatilis|ww03157
KF491576.1|Australothis volatilis
Australothis volatilis|ww03158
EU768897.1|Australothis volatilis
Australothis tertia|ww03156
KF390688.1|Australothis tertia|CCDB-15848-A12
KP688454.1|Australothis sp.
KP688453.1|Australothis sp.
Australothis rubrescens|ww03154
Australothis rubrescens|ww03152
KP688435.1|Australothis sp.
KP688452.1|Australothis sp.
KP688450.1|Australothis sp.
KP688449.1|Australothis sp.
KP688447.1|Australothis sp.
KP688434.1|Australothis sp.
KP688433.1|Australothis sp.
HQ951237.1|Australothis rubrescens|10ANIC-08279
Australothis rubrescens|ww03153
Australothis exopisso|ww06417
Australothis exopisso|ww06418
Australothis exopisso|ww06419
Australothis exopisso|ww03151
Australothis exopisso|ww03150
KF389201.1|Australothis exopisso|CCDB-15848-A11
Australothis exopisso|ww03149
KP688405.1|Australothis sp.
KP688408.1|Australothis sp.
KP688448.1|Australothis sp.
KP688446.1|Australothis sp.
KP688409.1|Australothis sp.
KP688411.1|Australothis sp.
EU768896.1|Australothis rubrescens
KP688407.1|Australothis sp.
KP688410.1|Australothis sp.
KP688451.1|Australothis sp.
KP688406.1|Australothis sp.
KF492621.1|Helicoverpa atacamae
KF492622.1|Helicoverpa atacamae
HQ951241.1|Helicoverpa punctigera|10ANIC-08284
HQ951242.1|Helicoverpa punctigera|10ANIC-08285
AY898747.1|Helicoverpa punctigera

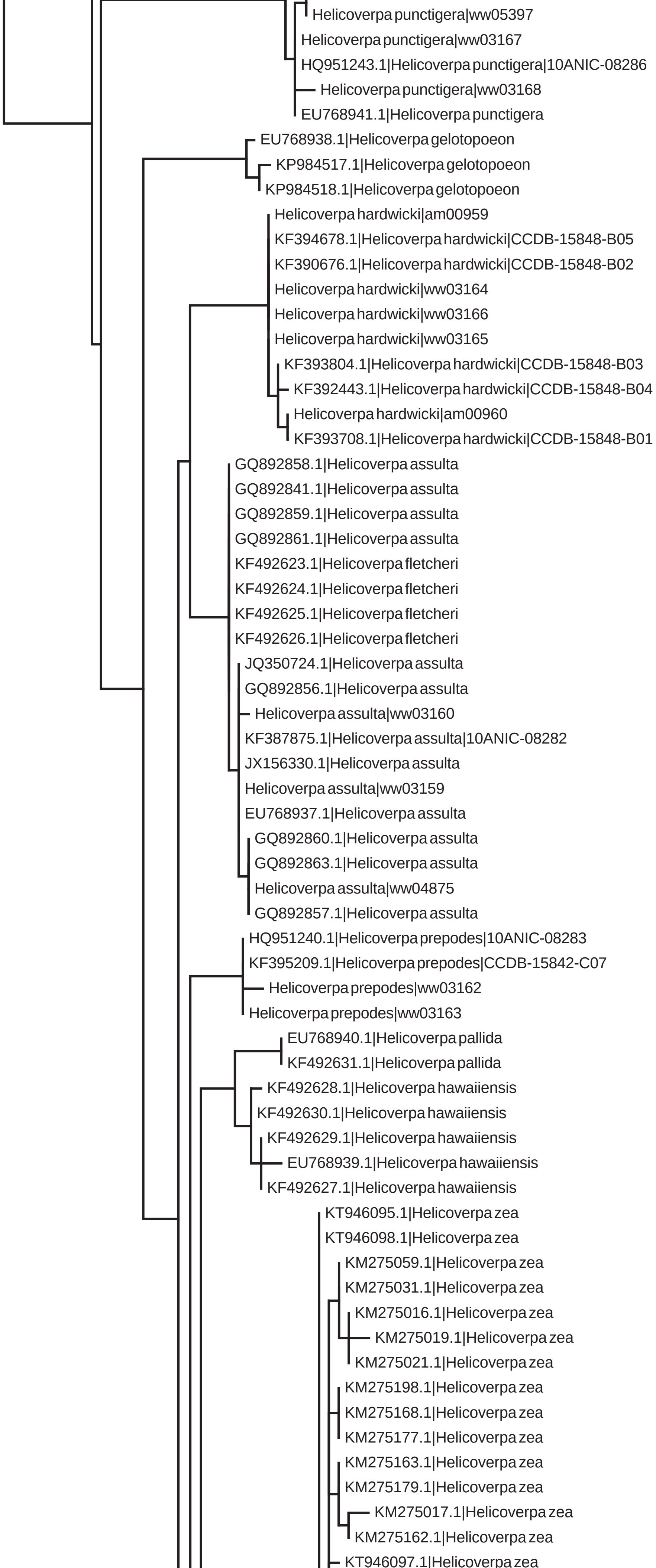
Helicoverpa punctigera|ww05397
Helicoverpa punctigera|ww03167
HQ951243.1|Helicoverpa punctigera|10ANIC-08286
Helicoverpa punctigera|ww03168
EU768941.1|Helicoverpa punctigera
EU768938.1|Helicoverpa gelotopoeon
KP984517.1|Helicoverpa gelotopoeon
KP984518.1|Helicoverpa gelotopoeon
Helicoverpa hardwicki|am00959
KF394678.1|Helicoverpa hardwicki|CCDB-15848-B05
KF390676.1|Helicoverpa hardwicki|CCDB-15848-B02
Helicoverpa hardwicki|ww03164
Helicoverpa hardwicki|ww03166
Helicoverpa hardwicki|ww03165
KF393804.1|Helicoverpa hardwicki|CCDB-15848-B03
KF392443.1|Helicoverpa hardwicki|CCDB-15848-B04
Helicoverpa hardwicki|am00960
KF393708.1|Helicoverpa hardwicki|CCDB-15848-B01
GQ892858.1|Helicoverpa assulta
GQ892841.1|Helicoverpa assulta
GQ892859.1|Helicoverpa assulta
GQ892861.1|Helicoverpa assulta
KF492623.1|Helicoverpa fletcheri
KF492624.1|Helicoverpa fletcheri
KF492625.1|Helicoverpa fletcheri
KF492626.1|Helicoverpa fletcheri
JQ350724.1|Helicoverpa assulta
GQ892856.1|Helicoverpa assulta
Helicoverpa assulta|ww03160
KF387875.1|Helicoverpa assulta|10ANIC-08282
JX156330.1|Helicoverpa assulta
Helicoverpa assulta|ww03159
EU768937.1|Helicoverpa assulta
GQ892860.1|Helicoverpa assulta
GQ892863.1|Helicoverpa assulta
Helicoverpa assulta|ww04875
GQ892857.1|Helicoverpa assulta
HQ951240.1|Helicoverpa prepodes|10ANIC-08283
KF395209.1|Helicoverpa prepodes|CCDB-15842-C07
Helicoverpa prepodes|ww03162
Helicoverpa prepodes|ww03163
EU768940.1|Helicoverpa pallida
KF492631.1|Helicoverpa pallida
KF492628.1|Helicoverpa hawaiiensis
KF492630.1|Helicoverpa hawaiiensis
KF492629.1|Helicoverpa hawaiiensis
EU768939.1|Helicoverpa hawaiiensis
KF492627.1|Helicoverpa hawaiiensis
KT946095.1|Helicoverpa zea
KT946098.1|Helicoverpa zea
KM275059.1|Helicoverpa zea
KM275031.1|Helicoverpa zea
KM275016.1|Helicoverpa zea
KM275019.1|Helicoverpa zea
KM275021.1|Helicoverpa zea
KM275198.1|Helicoverpa zea
KM275168.1|Helicoverpa zea
KM275177.1|Helicoverpa zea
KM275163.1|Helicoverpa zea
KM275179.1|Helicoverpa zea
KM275017.1|Helicoverpa zea
KM275162.1|Helicoverpa zea
KT946097.1|Helicoverpa zea

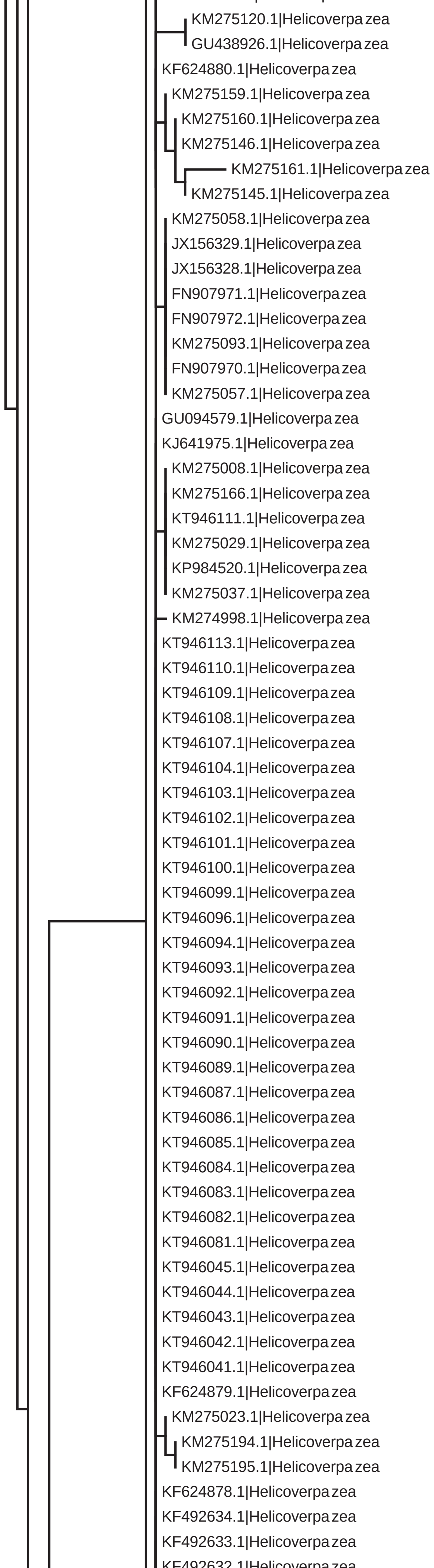
KM275120.1|Helicoverpa zea
GU438926.1|Helicoverpa zea
KF624880.1|Helicoverpa zea
KM275159.1|Helicoverpa zea
KM275160.1|Helicoverpa zea
KM275146.1|Helicoverpa zea
KM275161.1|Helicoverpa zea
KM275145.1|Helicoverpa zea
KM275058.1|Helicoverpa zea
JX156329.1|Helicoverpa zea
JX156328.1|Helicoverpa zea
FN907971.1|Helicoverpa zea
FN907972.1|Helicoverpa zea
KM275093.1|Helicoverpa zea
FN907970.1|Helicoverpa zea
KM275057.1|Helicoverpa zea
GU094579.1|Helicoverpa zea
KJ641975.1|Helicoverpa zea
KM275008.1|Helicoverpa zea
KM275166.1|Helicoverpa zea
KT946111.1|Helicoverpa zea
KM275029.1|Helicoverpa zea
KP984520.1|Helicoverpa zea
KM275037.1|Helicoverpa zea
KM274998.1|Helicoverpa zea
KT946113.1|Helicoverpa zea
KT946110.1|Helicoverpa zea
KT946109.1|Helicoverpa zea
KT946108.1|Helicoverpa zea
KT946107.1|Helicoverpa zea
KT946104.1|Helicoverpa zea
KT946103.1|Helicoverpa zea
KT946102.1|Helicoverpa zea
KT946101.1|Helicoverpa zea
KT946100.1|Helicoverpa zea
KT946099.1|Helicoverpa zea
KT946096.1|Helicoverpa zea
KT946094.1|Helicoverpa zea
KT946093.1|Helicoverpa zea
KT946092.1|Helicoverpa zea
KT946091.1|Helicoverpa zea
KT946090.1|Helicoverpa zea
KT946089.1|Helicoverpa zea
KT946087.1|Helicoverpa zea
KT946086.1|Helicoverpa zea
KT946085.1|Helicoverpa zea
KT946084.1|Helicoverpa zea
KT946083.1|Helicoverpa zea
KT946082.1|Helicoverpa zea
KT946081.1|Helicoverpa zea
KT946045.1|Helicoverpa zea
KT946044.1|Helicoverpa zea
KT946043.1|Helicoverpa zea
KT946042.1|Helicoverpa zea
KT946041.1|Helicoverpa zea
KF624879.1|Helicoverpa zea
KM275023.1|Helicoverpa zea
KM275194.1|Helicoverpa zea
KM275195.1|Helicoverpa zea
KF624878.1|Helicoverpa zea
KF492634.1|Helicoverpa zea
KF492633.1|Helicoverpa zea

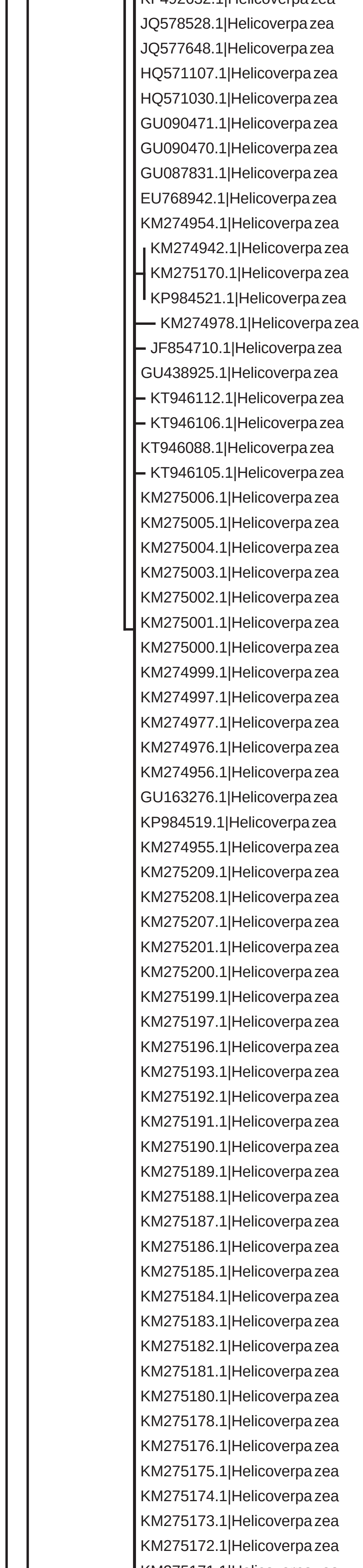
JQ578528.1|Helicoverpa zea
JQ577648.1|Helicoverpa zea
HQ571107.1|Helicoverpa zea
HQ571030.1|Helicoverpa zea
GU090471.1|Helicoverpa zea
GU090470.1|Helicoverpa zea
GU087831.1|Helicoverpa zea
EU768942.1|Helicoverpa zea
KM274954.1|Helicoverpa zea
KM274942.1|Helicoverpa zea
KM275170.1|Helicoverpa zea
KP984521.1|Helicoverpa zea
KM274978.1|Helicoverpa zea
JF854710.1|Helicoverpa zea
GU438925.1|Helicoverpa zea
KT946112.1|Helicoverpa zea
KT946106.1|Helicoverpa zea
KT946088.1|Helicoverpa zea
KT946105.1|Helicoverpa zea
KM275006.1|Helicoverpa zea
KM275005.1|Helicoverpa zea
KM275004.1|Helicoverpa zea
KM275003.1|Helicoverpa zea
KM275002.1|Helicoverpa zea
KM275001.1|Helicoverpa zea
KM275000.1|Helicoverpa zea
KM274999.1|Helicoverpa zea
KM274997.1|Helicoverpa zea
KM274977.1|Helicoverpa zea
KM274976.1|Helicoverpa zea
KM274956.1|Helicoverpa zea
GU163276.1|Helicoverpa zea
KP984519.1|Helicoverpa zea
KM274955.1|Helicoverpa zea
KM275209.1|Helicoverpa zea
KM275208.1|Helicoverpa zea
KM275207.1|Helicoverpa zea
KM275201.1|Helicoverpa zea
KM275200.1|Helicoverpa zea
KM275199.1|Helicoverpa zea
KM275197.1|Helicoverpa zea
KM275196.1|Helicoverpa zea
KM275193.1|Helicoverpa zea
KM275192.1|Helicoverpa zea
KM275191.1|Helicoverpa zea
KM275190.1|Helicoverpa zea
KM275189.1|Helicoverpa zea
KM275188.1|Helicoverpa zea
KM275187.1|Helicoverpa zea
KM275186.1|Helicoverpa zea
KM275185.1|Helicoverpa zea
KM275184.1|Helicoverpa zea
KM275183.1|Helicoverpa zea
KM275182.1|Helicoverpa zea
KM275181.1|Helicoverpa zea
KM275180.1|Helicoverpa zea
KM275178.1|Helicoverpa zea
KM275176.1|Helicoverpa zea
KM275175.1|Helicoverpa zea
KM275174.1|Helicoverpa zea
KM275173.1|Helicoverpa zea
KM275172.1|Helicoverpa zea

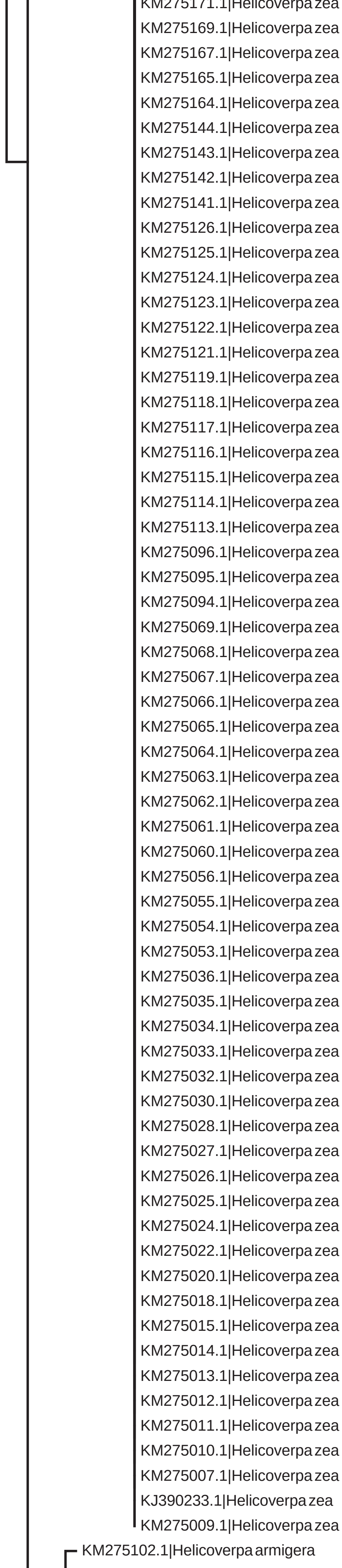

KM275171.1|Helicoverpa zea
KM275169.1|Helicoverpa zea
KM275167.1|Helicoverpa zea
KM275165.1|Helicoverpa zea
KM275164.1|Helicoverpa zea
KM275144.1|Helicoverpa zea
KM275143.1|Helicoverpa zea
KM275142.1|Helicoverpa zea
KM275141.1|Helicoverpa zea
KM275126.1|Helicoverpa zea
KM275125.1|Helicoverpa zea
KM275124.1|Helicoverpa zea
KM275123.1|Helicoverpa zea
KM275122.1|Helicoverpa zea
KM275121.1|Helicoverpa zea
KM275119.1|Helicoverpa zea
KM275118.1|Helicoverpa zea
KM275117.1|Helicoverpa zea
KM275116.1|Helicoverpa zea
KM275115.1|Helicoverpa zea
KM275114.1|Helicoverpa zea
KM275113.1|Helicoverpa zea
KM275096.1|Helicoverpa zea
KM275095.1|Helicoverpa zea
KM275094.1|Helicoverpa zea
KM275069.1|Helicoverpa zea
KM275068.1|Helicoverpa zea
KM275067.1|Helicoverpa zea
KM275066.1|Helicoverpa zea
KM275065.1|Helicoverpa zea
KM275064.1|Helicoverpa zea
KM275063.1|Helicoverpa zea
KM275062.1|Helicoverpa zea
KM275061.1|Helicoverpa zea
KM275060.1|Helicoverpa zea
KM275056.1|Helicoverpa zea
KM275055.1|Helicoverpa zea
KM275054.1|Helicoverpa zea
KM275053.1|Helicoverpa zea
KM275036.1|Helicoverpa zea
KM275035.1|Helicoverpa zea
KM275034.1|Helicoverpa zea
KM275033.1|Helicoverpa zea
KM275032.1|Helicoverpa zea
KM275030.1|Helicoverpa zea
KM275028.1|Helicoverpa zea
KM275027.1|Helicoverpa zea
KM275026.1|Helicoverpa zea
KM275025.1|Helicoverpa zea
KM275024.1|Helicoverpa zea
KM275022.1|Helicoverpa zea
KM275020.1|Helicoverpa zea
KM275018.1|Helicoverpa zea
KM275015.1|Helicoverpa zea
KM275014.1|Helicoverpa zea
KM275013.1|Helicoverpa zea
KM275012.1|Helicoverpa zea
KM275011.1|Helicoverpa zea
KM275010.1|Helicoverpa zea
KM275007.1|Helicoverpa zea
KJ390233.1|Helicoverpa zea
KM275009.1|Helicoverpa zea
KM275102.1|Helicoverpa armigera

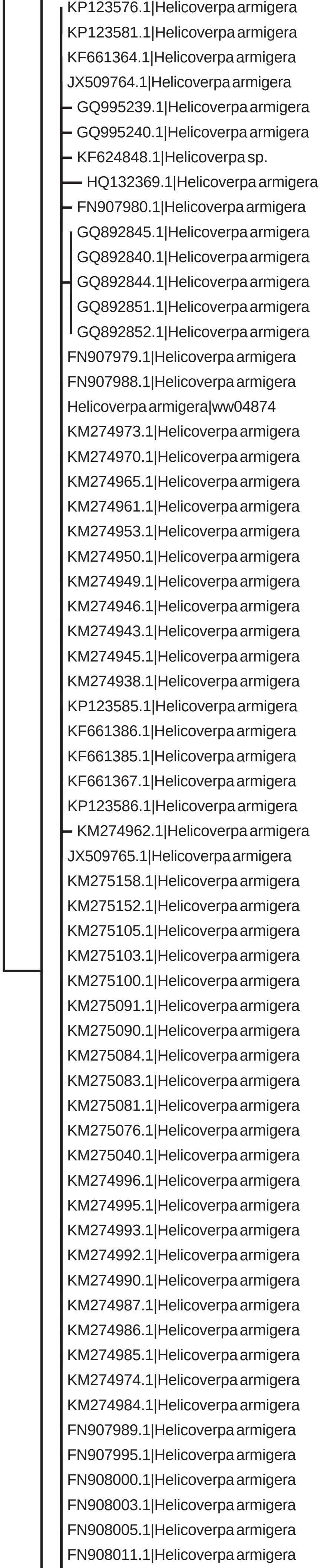
KP123576.1|Helicoverpa armigera
KP123581.1|Helicoverpa armigera
KF661364.1|Helicoverpa armigera
JX509764.1|Helicoverpa armigera
GQ995239.1|Helicoverpa armigera
GQ995240.1|Helicoverpa armigera
KF624848.1|Helicoverpa sp.
HQ132369.1|Helicoverpa armigera
FN907980.1|Helicoverpa armigera
GQ892845.1|Helicoverpa armigera
GQ892840.1|Helicoverpa armigera
GQ892844.1|Helicoverpa armigera
GQ892851.1|Helicoverpa armigera
GQ892852.1|Helicoverpa armigera
FN907979.1|Helicoverpa armigera
FN907988.1|Helicoverpa armigera
Helicoverpa armigera|ww04874
KM274973.1|Helicoverpa armigera
KM274970.1|Helicoverpa armigera
KM274965.1|Helicoverpa armigera
KM274961.1|Helicoverpa armigera
KM274953.1|Helicoverpa armigera
KM274950.1|Helicoverpa armigera
KM274949.1|Helicoverpa armigera
KM274946.1|Helicoverpa armigera
KM274943.1|Helicoverpa armigera
KM274945.1|Helicoverpa armigera
KM274938.1|Helicoverpa armigera
KP123585.1|Helicoverpa armigera
KF661386.1|Helicoverpa armigera
KF661385.1|Helicoverpa armigera
KF661367.1|Helicoverpa armigera
KP123586.1|Helicoverpa armigera
KM274962.1|Helicoverpa armigera
JX509765.1|Helicoverpa armigera
KM275158.1|Helicoverpa armigera
KM275152.1|Helicoverpa armigera
KM275105.1|Helicoverpa armigera
KM275103.1|Helicoverpa armigera
KM275100.1|Helicoverpa armigera
KM275091.1|Helicoverpa armigera
KM275090.1|Helicoverpa armigera
KM275084.1|Helicoverpa armigera
KM275083.1|Helicoverpa armigera
KM275081.1|Helicoverpa armigera
KM275076.1|Helicoverpa armigera
KM275040.1|Helicoverpa armigera
KM274996.1|Helicoverpa armigera
KM274995.1|Helicoverpa armigera
KM274993.1|Helicoverpa armigera
KM274992.1|Helicoverpa armigera
KM274990.1|Helicoverpa armigera
KM274987.1|Helicoverpa armigera
KM274986.1|Helicoverpa armigera
KM274985.1|Helicoverpa armigera
KM274974.1|Helicoverpa armigera
KM274984.1|Helicoverpa armigera
FN907989.1|Helicoverpa armigera
FN907995.1|Helicoverpa armigera
FN908000.1|Helicoverpa armigera
FN908003.1|Helicoverpa armigera
FN908005.1|Helicoverpa armigera
FN908011.1|Helicoverpa armigera

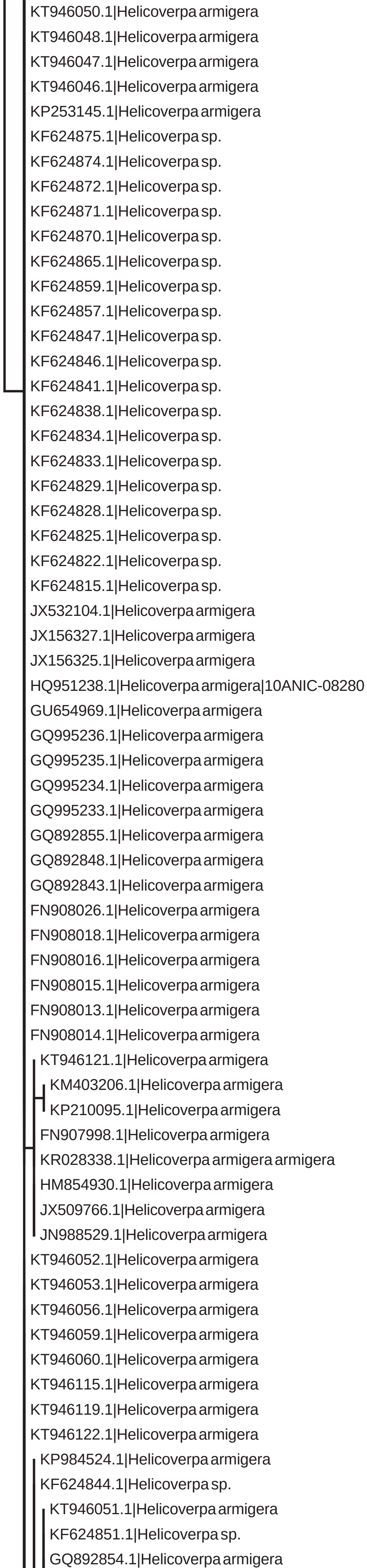

KT946050.1|Helicoverpa armigera
KT946048.1|Helicoverpa armigera
KT946047.1|Helicoverpa armigera
KT946046.1|Helicoverpa armigera
KP253145.1|Helicoverpa armigera
KF624875.1|Helicoverpa sp.
KF624874.1|Helicoverpa sp.
KF624872.1|Helicoverpa sp.
KF624871.1|Helicoverpa sp.
KF624870.1|Helicoverpa sp.
KF624865.1|Helicoverpa sp.
KF624859.1|Helicoverpa sp.
KF624857.1|Helicoverpa sp.
KF624847.1|Helicoverpa sp.
KF624846.1|Helicoverpa sp.
KF624841.1|Helicoverpa sp.
KF624838.1|Helicoverpa sp.
KF624834.1|Helicoverpa sp.
KF624833.1|Helicoverpa sp.
KF624829.1|Helicoverpa sp.
KF624828.1|Helicoverpa sp.
KF624825.1|Helicoverpa sp.
KF624822.1|Helicoverpa sp.
KF624815.1|Helicoverpa sp.
JX532104.1|Helicoverpa armigera
JX156327.1|Helicoverpa armigera
JX156325.1|Helicoverpa armigera
HQ951238.1|Helicoverpa armigera|10ANIC-08280
GU654969.1|Helicoverpa armigera
GQ995236.1|Helicoverpa armigera
GQ995235.1|Helicoverpa armigera
GQ995234.1|Helicoverpa armigera
GQ995233.1|Helicoverpa armigera
GQ892855.1|Helicoverpa armigera
GQ892848.1|Helicoverpa armigera
GQ892843.1|Helicoverpa armigera
FN908026.1|Helicoverpa armigera
FN908018.1|Helicoverpa armigera
FN908016.1|Helicoverpa armigera
FN908015.1|Helicoverpa armigera
FN908013.1|Helicoverpa armigera
FN908014.1|Helicoverpa armigera
KT946121.1|Helicoverpa armigera
KM403206.1|Helicoverpa armigera
KP210095.1|Helicoverpa armigera
FN907998.1|Helicoverpa armigera
KR028338.1|Helicoverpa armigera armigera
HM854930.1|Helicoverpa armigera
JX509766.1|Helicoverpa armigera
JN988529.1|Helicoverpa armigera
KT946052.1|Helicoverpa armigera
KT946053.1|Helicoverpa armigera
KT946056.1|Helicoverpa armigera
KT946059.1|Helicoverpa armigera
KT946060.1|Helicoverpa armigera
KT946115.1|Helicoverpa armigera
KT946119.1|Helicoverpa armigera
KT946122.1|Helicoverpa armigera
KP984524.1|Helicoverpa armigera
KF624844.1|Helicoverpa sp.
KT946051.1|Helicoverpa armigera
KF624851.1|Helicoverpa sp.
GQ892854.1|Helicoverpa armigera

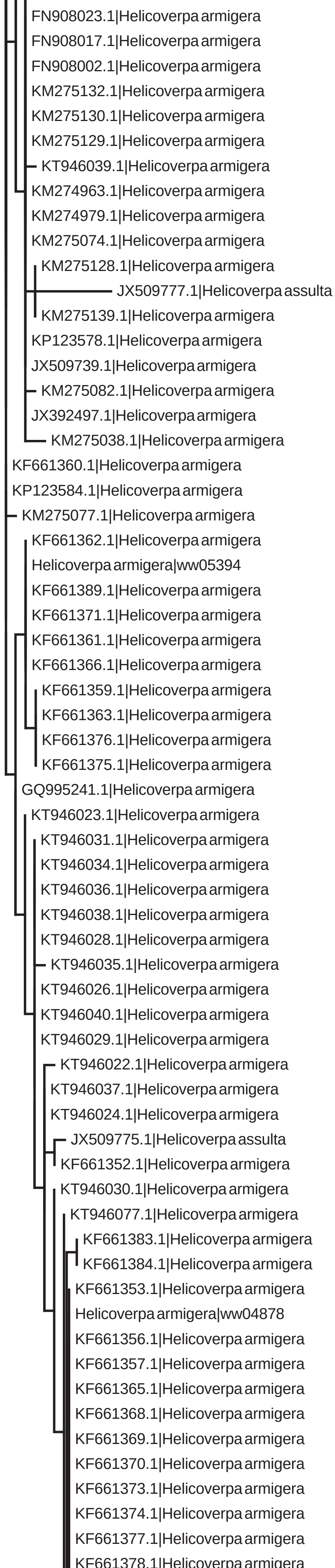
FN908023.1|Helicoverpa armigera
FN908017.1|Helicoverpa armigera
FN908002.1|Helicoverpa armigera
KM275132.1|Helicoverpa armigera
KM275130.1|Helicoverpa armigera
KM275129.1|Helicoverpa armigera
KT946039.1|Helicoverpa armigera
KM274963.1|Helicoverpa armigera
KM274979.1|Helicoverpa armigera
KM275074.1|Helicoverpa armigera
KM275128.1|Helicoverpa armigera
JX509777.1|Helicoverpa assulta
KM275139.1|Helicoverpa armigera
KP123578.1|Helicoverpa armigera
JX509739.1|Helicoverpa armigera
KM275082.1|Helicoverpa armigera
JX392497.1|Helicoverpa armigera
KM275038.1|Helicoverpa armigera
KF661360.1|Helicoverpa armigera
KP123584.1|Helicoverpa armigera
KM275077.1|Helicoverpa armigera
KF661362.1|Helicoverpa armigera
Helicoverpa armigera|ww05394
KF661389.1|Helicoverpa armigera
KF661371.1|Helicoverpa armigera
KF661361.1|Helicoverpa armigera
KF661366.1|Helicoverpa armigera
KF661359.1|Helicoverpa armigera
KF661363.1|Helicoverpa armigera
KF661376.1|Helicoverpa armigera
KF661375.1|Helicoverpa armigera
GQ995241.1|Helicoverpa armigera
KT946023.1|Helicoverpa armigera
KT946031.1|Helicoverpa armigera
KT946034.1|Helicoverpa armigera
KT946036.1|Helicoverpa armigera
KT946038.1|Helicoverpa armigera
KT946028.1|Helicoverpa armigera
KT946035.1|Helicoverpa armigera
KT946026.1|Helicoverpa armigera
KT946040.1|Helicoverpa armigera
KT946029.1|Helicoverpa armigera
KT946022.1|Helicoverpa armigera
KT946037.1|Helicoverpa armigera
KT946024.1|Helicoverpa armigera
JX509775.1|Helicoverpa assulta
KF661352.1|Helicoverpa armigera
KT946030.1|Helicoverpa armigera
KT946077.1|Helicoverpa armigera
KF661383.1|Helicoverpa armigera
KF661384.1|Helicoverpa armigera
KF661353.1|Helicoverpa armigera
Helicoverpa armigera|ww04878
KF661356.1|Helicoverpa armigera
KF661357.1|Helicoverpa armigera
KF661365.1|Helicoverpa armigera
KF661368.1|Helicoverpa armigera
KF661369.1|Helicoverpa armigera
KF661370.1|Helicoverpa armigera
KF661373.1|Helicoverpa armigera
KF661374.1|Helicoverpa armigera
KF661377.1|Helicoverpa armigera

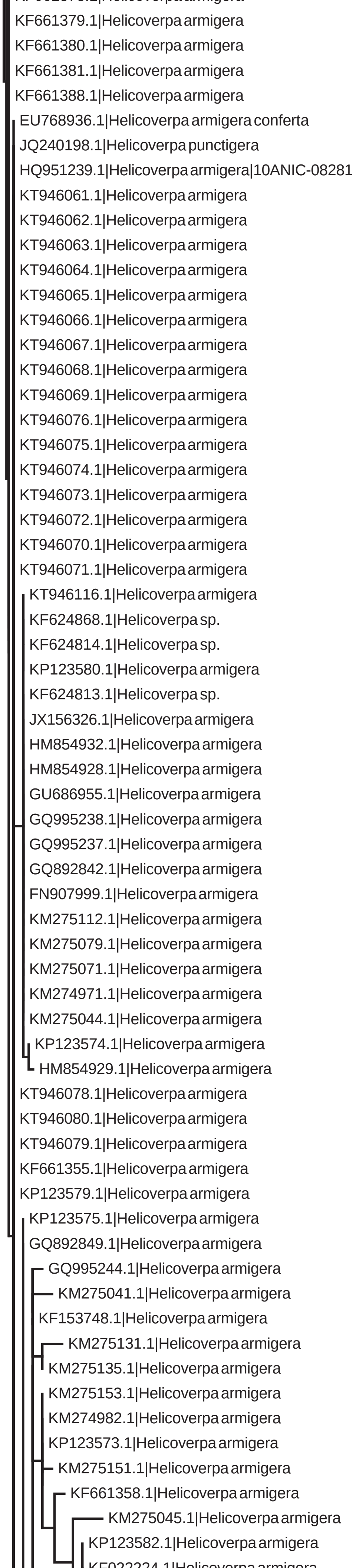
KF661379.1|Helicoverpa armigera
KF661380.1|Helicoverpa armigera
KF661381.1|Helicoverpa armigera
KF661388.1|Helicoverpa armigera
EU768936.1|Helicoverpa armigera conferta
JQ240198.1|Helicoverpa punctigera
HQ951239.1|Helicoverpa armigera|10ANIC-08281
KT946061.1|Helicoverpa armigera
KT946062.1|Helicoverpa armigera
KT946063.1|Helicoverpa armigera
KT946064.1|Helicoverpa armigera
KT946065.1|Helicoverpa armigera
KT946066.1|Helicoverpa armigera
KT946067.1|Helicoverpa armigera
KT946068.1|Helicoverpa armigera
KT946069.1|Helicoverpa armigera
KT946076.1|Helicoverpa armigera
KT946075.1|Helicoverpa armigera
KT946074.1|Helicoverpa armigera
KT946073.1|Helicoverpa armigera
KT946072.1|Helicoverpa armigera
KT946070.1|Helicoverpa armigera
KT946071.1|Helicoverpa armigera
KT946116.1|Helicoverpa armigera
KF624868.1|Helicoverpa sp.
KF624814.1|Helicoverpa sp.
KP123580.1|Helicoverpa armigera
KF624813.1|Helicoverpa sp.
JX156326.1|Helicoverpa armigera
HM854932.1|Helicoverpa armigera
HM854928.1|Helicoverpa armigera
GU686955.1|Helicoverpa armigera
GQ995238.1|Helicoverpa armigera
GQ995237.1|Helicoverpa armigera
GQ892842.1|Helicoverpa armigera
FN907999.1|Helicoverpa armigera
KM275112.1|Helicoverpa armigera
KM275079.1|Helicoverpa armigera
KM275071.1|Helicoverpa armigera
KM274971.1|Helicoverpa armigera
KM275044.1|Helicoverpa armigera
KP123574.1|Helicoverpa armigera
HM854929.1|Helicoverpa armigera
KT946078.1|Helicoverpa armigera
KT946080.1|Helicoverpa armigera
KT946079.1|Helicoverpa armigera
KF661355.1|Helicoverpa armigera
KP123579.1|Helicoverpa armigera
KP123575.1|Helicoverpa armigera
GQ892849.1|Helicoverpa armigera
GQ995244.1|Helicoverpa armigera
KM275041.1|Helicoverpa armigera
KF153748.1|Helicoverpa armigera
KM275131.1|Helicoverpa armigera
KM275135.1|Helicoverpa armigera
KM275153.1|Helicoverpa armigera
KM274982.1|Helicoverpa armigera
KP123573.1|Helicoverpa armigera
KM275151.1|Helicoverpa armigera
KF661358.1|Helicoverpa armigera
KM275045.1|Helicoverpa armigera
KP123582.1|Helicoverpa armigera

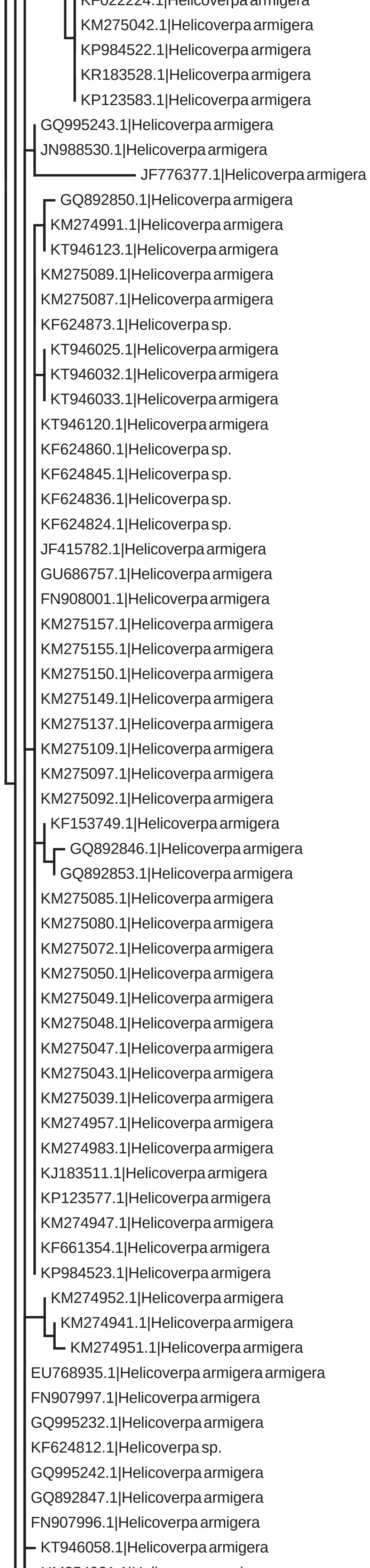
KM275042.1|Helicoverpa armigera
KP984522.1|Helicoverpa armigera
KR183528.1|Helicoverpa armigera
KP123583.1|Helicoverpa armigera
GQ995243.1|Helicoverpa armigera
JN988530.1|Helicoverpa armigera
JF776377.1|Helicoverpa armigera
GQ892850.1|Helicoverpa armigera
KM274991.1|Helicoverpa armigera
KT946123.1|Helicoverpa armigera
KM275089.1|Helicoverpa armigera
KM275087.1|Helicoverpa armigera
KF624873.1|Helicoverpa sp.
KT946025.1|Helicoverpa armigera
KT946032.1|Helicoverpa armigera
KT946033.1|Helicoverpa armigera
KT946120.1|Helicoverpa armigera
KF624860.1|Helicoverpa sp.
KF624845.1|Helicoverpa sp.
KF624836.1|Helicoverpa sp.
KF624824.1|Helicoverpa sp.
JF415782.1|Helicoverpa armigera
GU686757.1|Helicoverpa armigera
FN908001.1|Helicoverpa armigera
KM275157.1|Helicoverpa armigera
KM275155.1|Helicoverpa armigera
KM275150.1|Helicoverpa armigera
KM275149.1|Helicoverpa armigera
KM275137.1|Helicoverpa armigera
KM275109.1|Helicoverpa armigera
KM275097.1|Helicoverpa armigera
KM275092.1|Helicoverpa armigera
KF153749.1|Helicoverpa armigera
GQ892846.1|Helicoverpa armigera
GQ892853.1|Helicoverpa armigera
KM275085.1|Helicoverpa armigera
KM275080.1|Helicoverpa armigera
KM275072.1|Helicoverpa armigera
KM275050.1|Helicoverpa armigera
KM275049.1|Helicoverpa armigera
KM275048.1|Helicoverpa armigera
KM275047.1|Helicoverpa armigera
KM275043.1|Helicoverpa armigera
KM275039.1|Helicoverpa armigera
KM274957.1|Helicoverpa armigera
KM274983.1|Helicoverpa armigera
KJ183511.1|Helicoverpa armigera
KP123577.1|Helicoverpa armigera
KM274947.1|Helicoverpa armigera
KF661354.1|Helicoverpa armigera
KP984523.1|Helicoverpa armigera
KM274952.1|Helicoverpa armigera
KM274941.1|Helicoverpa armigera
KM274951.1|Helicoverpa armigera
EU768935.1|Helicoverpa armigera armigera
FN907997.1|Helicoverpa armigera
GQ995232.1|Helicoverpa armigera
KF624812.1|Helicoverpa sp.
GQ995242.1|Helicoverpa armigera
GQ892847.1|Helicoverpa armigera
FN907996.1|Helicoverpa armigera
KT946058.1|Helicoverpa armigera

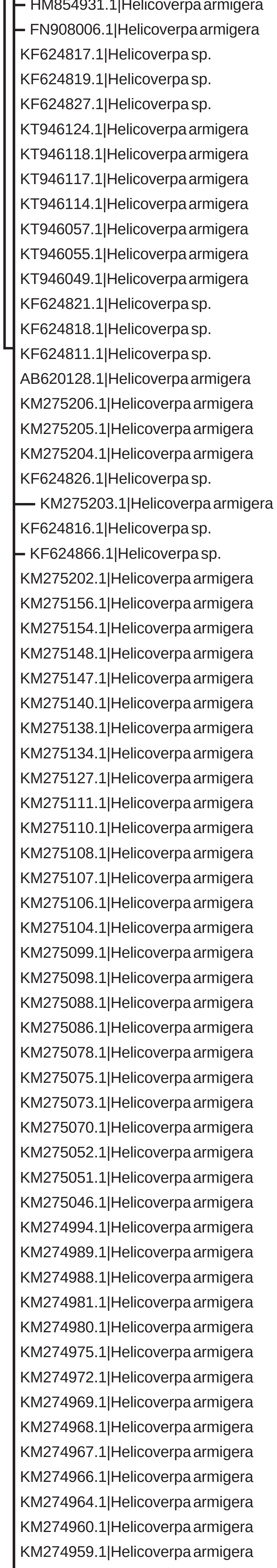
HM854931.1|Helicoverpa armigera
FN908006.1|Helicoverpa armigera
KF624817.1|Helicoverpa sp.
KF624819.1|Helicoverpa sp.
KF624827.1|Helicoverpa sp.
KT946124.1|Helicoverpa armigera
KT946118.1|Helicoverpa armigera
KT946117.1|Helicoverpa armigera
KT946114.1|Helicoverpa armigera
KT946057.1|Helicoverpa armigera
KT946055.1|Helicoverpa armigera
KT946049.1|Helicoverpa armigera
KF624821.1|Helicoverpa sp.
KF624818.1|Helicoverpa sp.
KF624811.1|Helicoverpa sp.
AB620128.1|Helicoverpa armigera
KM275206.1|Helicoverpa armigera
KM275205.1|Helicoverpa armigera
KM275204.1|Helicoverpa armigera
KF624826.1|Helicoverpa sp.
KM275203.1|Helicoverpa armigera
KF624816.1|Helicoverpa sp.
KF624866.1|Helicoverpa sp.
KM275202.1|Helicoverpa armigera
KM275156.1|Helicoverpa armigera
KM275154.1|Helicoverpa armigera
KM275148.1|Helicoverpa armigera
KM275147.1|Helicoverpa armigera
KM275140.1|Helicoverpa armigera
KM275138.1|Helicoverpa armigera
KM275134.1|Helicoverpa armigera
KM275127.1|Helicoverpa armigera
KM275111.1|Helicoverpa armigera
KM275110.1|Helicoverpa armigera
KM275108.1|Helicoverpa armigera
KM275107.1|Helicoverpa armigera
KM275106.1|Helicoverpa armigera
KM275104.1|Helicoverpa armigera
KM275099.1|Helicoverpa armigera
KM275098.1|Helicoverpa armigera
KM275088.1|Helicoverpa armigera
KM275086.1|Helicoverpa armigera
KM275078.1|Helicoverpa armigera
KM275075.1|Helicoverpa armigera
KM275073.1|Helicoverpa armigera
KM275070.1|Helicoverpa armigera
KM275052.1|Helicoverpa armigera
KM275051.1|Helicoverpa armigera
KM275046.1|Helicoverpa armigera
KM274994.1|Helicoverpa armigera
KM274989.1|Helicoverpa armigera
KM274988.1|Helicoverpa armigera
KM274981.1|Helicoverpa armigera
KM274980.1|Helicoverpa armigera
KM274975.1|Helicoverpa armigera
KM274972.1|Helicoverpa armigera
KM274969.1|Helicoverpa armigera
KM274968.1|Helicoverpa armigera
KM274967.1|Helicoverpa armigera
KM274966.1|Helicoverpa armigera
KM274964.1|Helicoverpa armigera
KM274960.1|Helicoverpa armigera
KM274959.1|Helicoverpa armigera

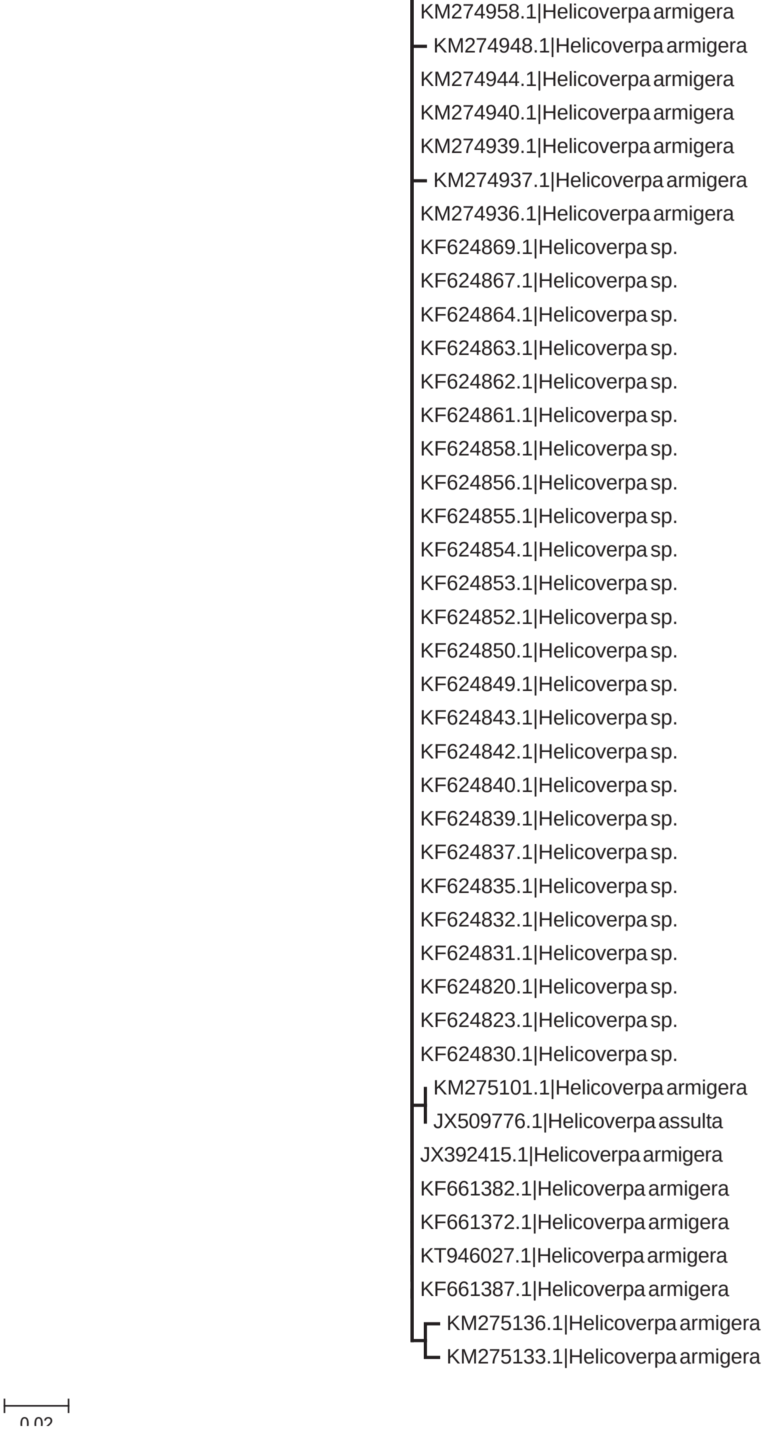
KM274958.1|Helicoverpa armigera
KM274948.1|Helicoverpa armigera
KM274944.1|Helicoverpa armigera
KM274940.1|Helicoverpa armigera
KM274939.1|Helicoverpa armigera
KM274937.1|Helicoverpa armigera
KM274936.1|Helicoverpa armigera
KF624869.1|Helicoverpa sp.
KF624867.1|Helicoverpa sp.
KF624864.1|Helicoverpa sp.
KF624863.1|Helicoverpa sp.
KF624862.1|Helicoverpa sp.
KF624861.1|Helicoverpa sp.
KF624858.1|Helicoverpa sp.
KF624856.1|Helicoverpa sp.
KF624855.1|Helicoverpa sp.
KF624854.1|Helicoverpa sp.
KF624853.1|Helicoverpa sp.
KF624852.1|Helicoverpa sp.
KF624850.1|Helicoverpa sp.
KF624849.1|Helicoverpa sp.
KF624843.1|Helicoverpa sp.
KF624842.1|Helicoverpa sp.
KF624840.1|Helicoverpa sp.
KF624839.1|Helicoverpa sp.
KF624837.1|Helicoverpa sp.
KF624835.1|Helicoverpa sp.
KF624832.1|Helicoverpa sp.
KF624831.1|Helicoverpa sp.
KF624820.1|Helicoverpa sp.
KF624823.1|Helicoverpa sp.
KF624830.1|Helicoverpa sp.
KM275101.1|Helicoverpa armigera
JX509776.1|Helicoverpa assulta
JX392415.1|Helicoverpa armigera
KF661382.1|Helicoverpa armigera
KF661372.1|Helicoverpa armigera
KT946027.1|Helicoverpa armigera
KF661387.1|Helicoverpa armigera
KM275136.1|Helicoverpa armigera
KM275133.1|Helicoverpa armigera
0.02
